# Time-dependent physical unclonable functions by long-lived triplet excitons in carbon dots

**DOI:** 10.1038/s41377-025-01940-9

**Published:** 2025-08-20

**Authors:** Yan-Wei Hu, Qing Cao, Shi-Yu Song, Yuan Sun, Ya-Chuan Liang, Wen-Bo Zhao, Chao-Fan Lv, Chong-Xin Shan, Kai-Kai Liu

**Affiliations:** 1 Henan Key Laboratory of Diamond Optoelectronic Materials and Devices, School of Physics and Laboratory of Zhongyuan Light, Zhengzhou University, Zhengzhou, China; 2 School of Electronics and Information, Zhengzhou University of Light Industry, Zhengzhou, China; 3 Institute of Quantum Materials and Physics Henan Academy of Sciences, Zhengzhou, 450046 China

**Keywords:** Optical spectroscopy, Imaging and sensing

## Abstract

Physical unclonable functions (PUFs), relying extensively on the random spatial distribution of block elements, are promising technology for generating unclonable cryptograph. Herein, we demonstrate time-dependent PUFs (TD-PUFs) by introducing carbon dots (CDs) with bright and long-lived triplet excitons as block elements. The constructed TD-PUFs evolve into multiple unclonable PUFs over time, effectively breaking the spatial limitation of transitional PUFs and increasing the complexity, making them much more difficult to be attacked. This temporal evolution introduces an additional layer of security, as the dynamic nature of TD-PUFs makes it increasingly challenging for adversaries to predict or replicate their states. We have developed pixel matrix function (PMF) to describe the evolution process of the TD-PUFs, enabling a detailed analysis of the dynamic behavior and unique security features. In addition, we exhibit a TD-PUFs painting (30 × 40 cm^2^) by an etching technology where the primary structures of the panting undergo a transformation over time, driven by the varying triplet exciton lifetimes of the CDs. The proposed concept of TD-PUFs overcome their spatial limitations and increase the complexity, making the PUF labels more difficulty to be cracked.

## Abstract (web summary)

Concept and application diagram based on TD-PUF.

## Graphical abstract

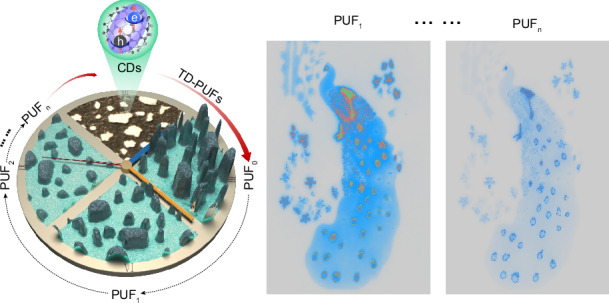

## Introduction

Physical Unclonable Functions (PUFs) derive their uniqueness from the intrinsic randomness within microscopic material structures, making them unclonable in subsequent productions. These substances respond accordingly when exposed to specific challenges such as light, electricity, or magnetism, and the one-to-one correspondence between these challenges and responses can be called unique challenge-response pair^1,2^. The first conceptual PUF was realized by optically exciting a three-dimensional object with a random physical structure inside, as introduced by Ravikanth Pappu and colleagues in 2002^3^. Since then, PUFs have received widespread attention and research due to their simple and low-cost preparation methods and strong anti-counterfeiting and encryption capabilities. According to their diverse challenges sources and application scenarios, PUFs can be divided into three major categories: (1) Silicon-based PUFs, challenged by electric fields and symbiotic with electronic devices^4^. The silicon PUFs are one of best-known subset of PUFs, and they are usually a part of an integrated circuit. The reading method for this type of PUFs is contact based, resulting in poor stability and limited application scenarios. (2) PUFs challenged by magnetic fields, such as magnetic simulation PUFs^5,6^, offer potential solutions. However, the intricate preparation process and the associated high costs persist as significant challenges. (3) Optical PUFs, utilizing light as a challenge have emerged as a significant development in recent years. Variants like edible PUFs^7^, flexible PUFs^8–10^, and luminescent PUFs^11–14^ have been widely used for anti-counterfeiting and high-level encryption. However, it is worth noting that most of these PUFs are based on spatial dimensions, which will limit the further improvement of PUF encoding capabilities. Im et al. prepared four-dimensional PUF (4D)^15^ by utilizing the continuous luminescence characteristics of phosphorescent crystals^16^, and Nocentini et al. exploits reconfigurable photo-responsive materials to create a multilevel PUF^17^. These methods can build more complex systems by increasing the dimensions of PUF, thereby increasing the anti-counterfeiting encryption capabilities of PUF. However, this study did not address the temporal evolution aspect of PUFs.

Triplet excitons, composed of coupled electron and hole pairs with parallel spins, yield photons containing observable time information due to spin-forbidden transition in accordance with the Pauli exclusion principle^18^. These long-lived emissive states are the foundation of phosphorescence, a phenomenon that has been investigated since the 1950s when Peter Pringsheim and colleagues began studying fluorescence and phosphorescence^19^. Later, Charles F. Förster and collaborators identified phosphorescence in plastics^20^. In recent years, the generation of photons from the radiative recombination of triplet excitons in organic materials^21^, carbon dots (CDs)^22–26^, and other metal-based complex materials^27–29^ have been widely achieved through the collaborative efforts of physicists, chemists, materials scientists, and other researchers. Generally, heteroatoms doping has been widely employed as an effective strategy to promote the formation of triplet excitons, while confinement serves as an effective method to inhibit their dissipation, facilitating the realization of triplet exciton emission^30^. The radiative transition rates from triplet excitons are typically slow due to the ‘spin-forbidden’ nature, leading to emission with lifetimes typically extending microseconds. Specially, the lifetimes of triplet exciton emission in CDs have been extended beyond tens of seconds, and with the emission of triplet excitons in CDs even achieved in aqueous solution, previously considered impossible^31,32^. The bright and long-lived triplet excitons, incorporating temporal information, serve as ideal candidates for TD-PUFs. CDs characterized by their eco-friendly nature, low cost, and ease of preparation, represent excellent platforms for utilizing triplet excitons in constructing TD-PUFs.

In previous work, we achieved colorful, bright, and long-lasting triplet exciton emission in CDs through conjugated size and confinement engineering^33^. In this work, we advance PUFs to TD-PUFs by leveraging the slow photon effects generated from triplet excitons in CDs, breaking the spatial limitation of traditional PUFs. We partitioned the TD-PUFs into a two-dimensional matrix and subsequently established a matrix function to depict the evolution of TD-PUFs over time by solving the boundary conditions and setting the lifetimes of triplet excitons in the CDs. Furthermore, the coding capacity of the TD-PUFs can be extended from 2^2,250,000^ to 255^2,250,000^, significantly increasing the complexity of cryptographic primitives. In addition, a multiple-coding painting featuring TD-PUFs has been developed, wherein the primary structures undergo a transformation over time. This transition is propelled by the colorful and time-dependent triplet excitons within the CDs, imparting dynamic visual properties that bolster artwork’s uniqueness and security attributes.

## Results

### Fabrication of TD-PUFs by populating triple excitons in CDs

**Fig. 1:**
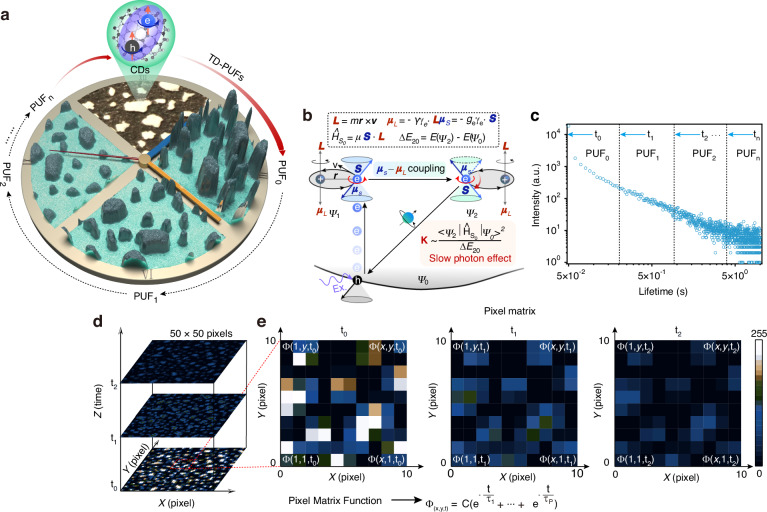
**Schematic diagram of TD-PUFs based on CDs with triplet excitons and the corresponding pixel matrix function**. **a** A schematic diagram illustrating the evolution of the TD-PUFs over time. **b** The population dynamics of triplet state electrons, and the slow photon effect caused by the spin-forbidden transition. The symbols ‘*L*’ and ‘*μ*_*L*_’ represent orbital angular momentum vector and its corresponding magnetic momentum vector, ‘*r*’ is orbit radius; The symbols ‘*S*’ and ‘*μ*_*S*_’ represent spin angular momentum vector and the its magnetic momentum; ${\varPsi }_{0}$, ${\varPsi }_{1}$, ${\varPsi }_{2}$ represent the ground state electron wavefunction, singlet state electron wavefunction, and triplet state electron wavefunction; ‘*K*’ and ‘${\Delta E}_{20}$’ represent the transition rate and energy split between electrons in ${\varPsi }_{2}$ and ${\varPsi }_{0}$, and ${\hat{H}}_{{so}}$ is the spin-orbit coupling Hamiltonian operator. **c** Phosphorescence decay curves (lifetime) under 365 nm excitation at room temperature. The lifetime curves are sliced into i_n_tervals with time units t_0_, t_1_, t_2_ to t_n_, and PUF_0_, PUF_1_, PUF_2_ to PUF_n_ can be generated in these intervals. **d** Grayscale value disribution diagram of TD-PUFs at time *t*_0_, *t*_1_ and *t*_2_ (50 × 50 pixels). **e** Enlarged matrix view (10 × 10 pixels) illustrating detailed evolution process of the TD-PUFs over time, and the constructed pixel matrix function is listed at the bottom

The variation in quantity and lifetime of CDs within each pixel of TD-PUFs contributes to the diverse variations observed in TD-PUFs over time, and the schematic diagram of TD-PUFs is shown in Fig. 1. The TD-PUFs was constructed using randomly distributed phosphorescent CDs as building block, and the reading process consists of three components: challenge, response, and digitization (Supplementary Fig. S1). Three kinds of CD powders, each with different sizes from 0.5 mm to 1.4 mm, were selected for the construction of TD-PUFs through different sieve meshes, as depicted in Supplementary Fig. S2. Subsequently, these different kinds of CDs were randomly mixed together to prepared TD-PUFs, the detailed process is illustrated in Supplementary Fig. S3. After excitation, the TD-PUFs will evolve into different PUFs over time following the removal of light source, owing to the prolonged luminescent properties of CDs. The newly generated PUFs exhibit significant differences attribute to variation in both quantity and lifetimes of the CDs within each pixel of the TD-PUFs, as illustrated in Fig. 1a. In the entire TD-PUFs system, the quantity of the derived PUFs is determined by the time interval, and the time interval can span from nanosecond to second. Therefore, the lifetime and phosphorescence intensity of CDs play a pivotal role in determining the efficacy of TD-PUFs. Specifically, these properties are governed by spin-orbit coupling (SOC)^29,34^ and non-radiative recombination processes. SOC facilitates intersystem crossing (ISC), allowing singlet excitons to transition into long-lived triplet states^34^. Due to the spin-forbidden nature of the triplet-to-singlet transition, the radiative decay is significantly prolonged, as illustrated in Fig. 1b. To enhance this process, exotic atoms were incorporated into the CD structure^35^, increasing SOC strength and thereby extending triplet-state lifetime. This mechanism has been extensively studied in our previous research^36^. Under 365 nm excitation, the luminescence of CDs extends up to 10 s, as shown in Fig. 1c. Detailed luminescent properties of CDs are characterized in Supplementary Figs. S4 and S5. TD-PUFs comprised of PUF_0_, PUF_1_, PUF_2_ and continuing up to PUF_n_, can be systematically constructed using the time slice technique, with the time interval determined by the shutter frequency.

To depict the evolution of TD-PUFs clearly in both temporal and spatial dimensions, the PUFs at time *t*_0_, *t*_1_ and *t*_2_ within the same spatial dimension are extracted, as illustrated in Fig. 1d. Here, the X-axis and Y-axis represent the spatial coordinates of the TD-PUFs, while the Z-axis represents temporal dimension. As time progresses, the TD-PUFs exhibit diverse configurations, and each distinct from the others. TD-PUFs (10 × 10 pixels) at time t_0_, t_1_ and t_2_ were selected to illustrate the variances and interconnections between them, as shown in Fig. 1e. For clarity, the PUFs were digitized and labeled as a two-dimensional pixel matrix $$\left[\begin{array}{ccc}{\Phi }_{({1,y},t)} & \cdots & {\Phi }_{(x,y,t)}\\ \vdots & \ddots & \vdots \\ {\Phi }_{(1,1,t)} & \cdots & {\Phi }_{(x,1,t)}\end{array}\right]$$, Where ${\Phi }_{(x,y,t)}$ represent value (here is gray value obtained by pseudo color transformation) of pixels, at moment ‘t’. For instance, the matrix of a PUF with 10 × 10 pixels at *t* = 1 s is denoted as $$\left[\begin{array}{ccc}{\Phi }_{(\mathrm{1,10,1})} & \cdots & {\Phi }_{(\mathrm{10,10,1})}\\ \vdots & \ddots & \vdots \\ {\Phi }_{(\mathrm{1,1,1})} & \cdots & {\Phi }_{(\mathrm{10,1,1})}\end{array}\right]$$. Therefore, any PUFs in this work can be represented as a Pixel Matrix Function (PMF), and the formula is as follows:

1 $${\varPhi }_{(x,y,t)}=\psi \left(x,y\right)T\left(t\right)$$

Here, $\psi \left(x,y\right)$ represents initial value at coordinate (x, y), and $T(t$) represents the lifetime of CDs in the pixel. Since the $\psi \left(x,y\right)$ does not change with time, it can be uniformly considered as equal to a constant *A*. Therefore, the new functional relationship can be expressed as:

2 $${\varPhi }_{(x,y,t)}={\rm{A}}T\left(t\right)$$

The time-dependent function for $T\left(t\right)$ can be expressed as:

3 $$T\left(t\right)={\rm{B}}\left({e}^{\frac{-t}{{\tau }_{1}}}+{e}^{\frac{-t}{{\tau }_{2}}}+\ldots +{e}^{\frac{-t}{{\tau }_{P}}}\right)$$

Where *B* represents the fitting coefficient, and *τ*_P_ denotes the fitted lifetimes of the CDs. Finally, the PMF description for the constructed TD-PUFs with respect to time is expressed as:

4 $${\varPhi }_{(x,y,t)}={\rm{C}}\left({e}^{\frac{-t}{{\tau }_{1}}}+{e}^{\frac{-t}{{\tau }_{2}}}+\ldots +{e}^{\frac{-t}{{\tau }_{P}}}\right)$$

Where *C* is the product of *A* and *B*. In different TD-PUFs, the specific value of *C* can be determined by calculating the boundary conditions at *t* = 0.

### The randomness of TD-PUFs over time

**Fig. 2:**
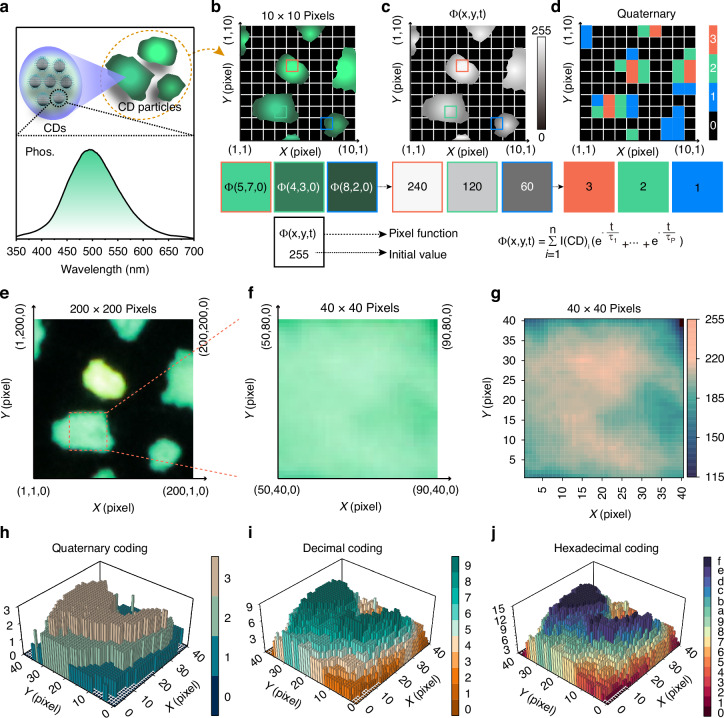
**Construction PMF through boundary condition**. **a** The illustration diagram of CD particles through assembly process, and the corresponding phosphorescence spectrum. **b** The optical photo simulating the response of the TD-PUFs, consisting 10 × 10 pixels. **c** Corresponding gray-scale picture and (**d**) quaternary coding picture. **e** The response of the TD-PUFs, consisting of 200 × 200 pixels. **f** The response map and (**g**) the corresponding grayscale image of the TD-PUFs with 40 × 40 pixels by intercepting square region spanning coordinates (50, 40) to (90, 80) at t = 0 s. **h**–**j** Corresponding quaternary coding, decimal coding and hexadecimal coding of the TD-PUFs with 40 × 40 pixels area

The differences in shape and size of CD particles result from the random assembly process of individual CDs, whereas the size of the CDs is ~5 nm while that of the CD particles is in the range of 0.2–1.5 mm (Fig. 2a and Supplementary Fig. S6). At room temperature, the phosphorescence wavelength peak of the CDs locates at around 500 nm under the optimal excitation of 365 nm. To evaluate the environmental robustness of the TD-PUF system during transportation, the CDs were subjected to systematic stability assessments under a range of conditions, including extreme temperatures (from –100 °C to +70 °C), varying relative humidity levels (11% to 84%), different atmospheric environments (air, argon, nitrogen, oxygen, and hydrogen), as well as extended storage durations (0, 30, and 180 days). As shown in Supplementary Figs. S7–S10, both the photoluminescence (PL) and phosphorescence signals of the CDs exhibited excellent stability across all tested conditions. By utilizing the shapes, phosphorescence and lifetime of CD particles, the diagram of TD-PUFs after the challenge is illustrated in Fig. 2b. The differences in the quantities of CDs within a pixel endow the pixel with varying luminescence intensities and lifetimes. To accurately construct a PMF for describing TD-PUFs, we need to determine the value of the constant *C* that is the summation of CD luminescence intensity within a pixel, which can be obtained by converting luminescence intensity to gray value. Three initial pixel values at *t* = 0 marked as (5, 7, 0), (4, 3, 0), and (8, 2, 0) were taken as examples to illustrate the construction process of the PMF, as depicted in the bottom of Fig. 2b. The luminescence image of the TD-PUFs was converted into grayscale image (Fig. 2c, top). The gray values of the three calibrated pixels were determined to be 240, 120, and 60, respectively (Fig. 2c, bottom). By employing a specific threshold, the grayscale image can be digitized into quaternary coding (Fig. 2d, top), wherein the bit values assigned to the three pixels were 3, 2, and 1, respectively (Fig. 2d, bottom). As time progresses, the gray value changes due to the variations in luminescence intensity, and the time *t* can be given by fitting the decay curves of triplet excitons. Thus, the PMF described for TD-PUFs based the triplet exciton emission can be constructed by solving boundary condition and fitting luminescence decay curves. The formula of PMF is shown as follows:

5 $${\varPhi }_{(x,y,t)}=\mathop{\sum }\limits_{i=1}^{n}{{\rm{I}}({\rm{CD}})}_{i}\left({e}^{\frac{-t}{{\tau }_{1}}}+{e}^{\frac{-t}{{\tau }_{2}}}+\ldots +{e}^{\frac{-t}{{\tau }_{P}}}\right)$$

Where ‘*I*’ represents the luminescence intensity of a CD, and ‘*i*’ is the number of CDs within a pixel. To experimentally verify this rule, TD-PUFs with 1500 × 1500 pixels were demonstrated and converting it into binary coding and quaternary coding. For clarity, the partial magnified TD-PUF consisting of 200 × 200 pixels was extracted for analysis (Fig. 2e), the global TD-PUFs and the corresponding binary and quaternary coding maps are shown in Supplementary Fig. S11. Additionally, a region of 40 × 40 pixels, intercepted from the mark square region spanning coordinates (50, 40) to (90, 80) at t = 0 s, is shown in Fig. 2f. Figure 2g shows the complete gray value picture of the TD-PUFs, displaying random gray values across different pixels, a series of values C for PMF can thus obtained. The corresponding quaternary coding, decimal coding and hexadecimal coding are shown in Fig. 2h–j, respectively, exhibiting their strong coding capability.

### Performance characterization of the TD-PUFs

We conducted a comprehensive security analysis of TD-PUFs coding from three aspects: coding capacity, uniqueness, and repeatability. These performance indicators are usually measured using Hamming distance (HD) or fractional Hamming distance (Fractional-HD) (Supplementary Fig. S12)^37,38^. The coding of a PUF can be likened to a key, and the coding capability represents the number of permutations and combinations of all keys within a PUF^39–41^. Therefore, the theoretical coding capacity of a PUF can be calculated as *M* = *A* * *B*, where M is the coding capacity, ‘*A*’ represents the number of responses per pixel, and ‘*B*’ represents the total number of pixels in the PUF^42–44^. For binary coding, with two possible values (0 and 1), and quaternary coding, with four possible values (0, 1, 2, and 3), the coding capacities are calculated accordingly. For example, a PUF with 1500 × 1500 pixels (2,250,000 bits) would have coding capacities of 2^2,250,000^ for binary coding and 4^2,250,000^ for quaternary coding, respectively. However, this is the maximum coding capacity that can be produced in theory. In fact, when calculating the coding capacity, the information entropy of the image needs to be taken into account, which will affect the coding capacity of TD-PUFs^37^. Here, the information entropy of TD-PUFs is defined as:

6 $$H=-\mathop{\sum }\limits_{i=1}^{B}p\left({x}_{i}\right){log }_{2}p\left({x}_{i}\right)$$

Here $H$ is the information entropy, ${x}_{i}$ is each pixel value in the image, $p({x}_{i})$ is the probability distribution of pixel value ${x}_{i}$ (the probability that the pixel value appears in the image), and B is the number of pixels in the image. Therefore, the actual coding capacities of PUFs can be expressed as M’ for binary images, where M’ = *H* *×* *B*.

The coding capacities of PUFs with different pixel counts are shown in Supplementary Table S1 in detail. Another important feature is uniqueness, which refers to the ability of keys within a PUF to resist duplication. This uniqueness can be quantified using Hamming inter-distance, which refers to the minimum number of substitutions required to transform one row of binary coding keys into another^45,46^. The formula for measuring uniqueness of a PUF is as follows:

7 $$U=\frac{2}{N(N-1)}\mathop{\sum }\limits_{i=1}^{N-1}\mathop{\sum }\limits_{j=i+1}^{N}\frac{{HD}({P}_{i},{P}_{j})}{L}$$

Where *U* is the value of the Hamming inter-distance between different PUFs, *P*_i_ and *P*_j_ are L-bit keys of the *i*th and *j*th rows among *N* rows. For example, a PUF with 1500 × 1500 pixels (1500-bit key with 1500 rows) can generate 1,124,250 results (${C}_{1500}^{2}$) of comparison. The repeatability can be quantified by the Hamming intra-distance, which refers to the number of different binary coding measured twice for the same PUF^47,48^. The repeatability of a PUF label can be defined as follows:

8 $$R=\frac{1}{T}\mathop{\sum }\limits_{t=1}^{T}\frac{{HD}({P}_{i},{P}_{i,t})}{L}\times 100 \%$$

Where *R* is the value of Hamming intra-distance between the same PUF measured at different times, *P*_i_ is the original L-bit reference key and *P*_i,t_ is the key extracted from the same PUF label measured at different time-point (t) among T measurements. The theoretical value of Hamming intra-distance is zero if a PUF has high stability. In addition, similarity index (*I*) is used to detect the degree of similarity between two PUFs^49,50^, which can provide a simple proof that a TD-PUF gradually generates new PUFs over time. The ‘*I*’ is given by:

9 $${\rm{I}}=\frac{C}{B}\times 100 \%$$

Where *C* is the same number of pixels between two PUFs and *B* is the total number of pixels.

**Fig. 3:**
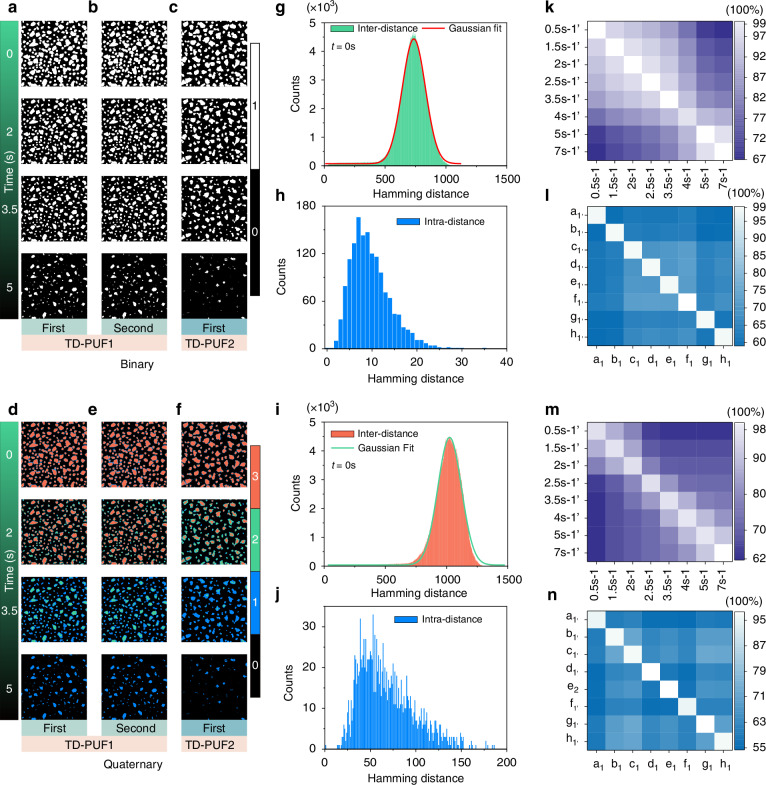
**Performance of the TD-PUFs**. The binary coding map of the TD-PUF_1_ measured for first (**a**) and second (**b**) times at different times. **c** The binary coding maps of TD-PUF_2_ measured at different times. The quaternary coding map of the TD-PUF_1_ measured for first (**d**) and second times (**e**) at different times. **f** The quaternary coding maps of TD-PUF_2_ measured at different times. **g** The Hamming inter-distance of the TD-PUF_1_ by measured at *t* = 0 s. **h** The Hamming intra-distance of the TD-PUF_1_ measured twice at *t* = 0 s. **i** The Hamming inter-distance of TD-PUF_1_ the with quaternary coding measured at *t* = 0 s. **j** The Hamming intra-distance of the TD-PUF_1_ with quaternary coding measured twice at *t* = 0 s. **k** Similarity statistical image of TD-PUF_1_ with binary coding measured twice at eight different moments. **l** Similarity statistical image of eight different TD-PUFs with binary coding measured twice at the *t* = 0 s. **m** Similarity statistical image of TD-PUF_1_ with quaternary coding twice at eight different moments. **n** Similarity statistical image for eight different TD-PUFs with quaternary coding measured twice at *t* = 0 s

The security performance investigation of TD-PUFs consisting of different CD particle sizes is shown in Fig. 3, three TD-PUFs containing a single CD particle size distribution from 0.5 mm to 1.4 mm are shown in Supplementary Figs. S13 and S14. The TD-PUFs constructed by different CD particle sizes have higher complexity and safety, thus two TD-PUFs consisting different CD particle sizes named as TD-PUF_1_ and TD-PUF_2_, were measured at different times, and then they were converted into binary coding and quaternary coding for analysis, as shown in Fig. 3a–f. Figures 3a, b display the binary coding results of two consecutive measurements of TD-PUF_1_, while Fig. 3c exhibits the measurement results of TD-PUF_2_. Uniformity result measured at time *t* = 0 (‘1’ bit as a percentage of the total bits in PUF) indicates that the TD-PUF_1_ and TD-PUF_2_ have good uniform (Supplementary Table S2). As time passed, the number of ‘0’ bits in the TD-PUF gradually increased, resulting in reduced uniformity, which is an unclonable characteristic of TD-PUFs. Figure 3d–f present the results of quaternary coding obtained using the same method, and they also exhibit a good uniformity (Supplementary Table S2). Subsequently, we conducted uniqueness analysis of the TD-PUFs through binary and quaternary coding at t = 0 s, as shown in Fig. 3g, i. According to theoretical expectations, the spacing outcomes adhere to the binomial distribution law, with a Hamming spacing of 750 exhibiting the highest probability of occurrence (representing half the total number of bits)^44,45^. Nonetheless, due to variations in the proportions of ‘0’ and ‘1’ bits within the PUFs, certain discrepancies were observed in the actual results. For example, at t = 0 s, the Hamming inter-distance statistics of TD-PUF_1_ show that the probability of occurrence is highest at 725, and this value decreases to 665 at t = 2 s (Fig. 3g and Supplementary Fig. S15). Figure 3h (binary) and Fig. 3j (quaternary) display the intra-distance values of the same PUF measured twice at *t* = 0 s. Similarly, the intra-distance comparison results at other time points are shown in Supplementary Fig. S16 (binary) and Supplementary Fig. S17 (quaternary). To eliminate contingency, we selected eight PUFs and measured them twice at t = 0.5 s for analysis and comparison, as shown in Supplementary Fig. S18 (binary). Notably, the measurement results of the same PUF measured at the same time exhibit remarkable repeatability. Furthermore, we performed two measurements on the TD-PUFs from *t* = 0.5 s to *t* = 7 s, resulting in 64 similarity index values obtained through pair-to-pair comparisons. The corresponding binary and quaternary statistical results are presented in Fig. 3k, m, indicating high repeatability and uniqueness. In addition, by changing the time interval between each PUF in TD-PUF, the crosstalk problem can be reduced, thereby improving the repeatability between PUFs (Supplementary Fig. S19). We also conducted measurements on eight different PUFs with 1500 × 1500 pixels, comparing the results of two measurements at *t* = 0 s, yielding 64 similarity index values, as shown in Fig. 3l, n. Supplementary Table S3 illustrates the 64 calculated similarity indexes for TD-PUFs by binary and quaternary coding. The comparison result indicates that the same PUF measured twice at the same moment exhibits a high similarity index, while low similarity index values were observed for different PUFs and the same PUF measured at different moments.

### Time effect in TD-PUFs

**Fig. 4:**
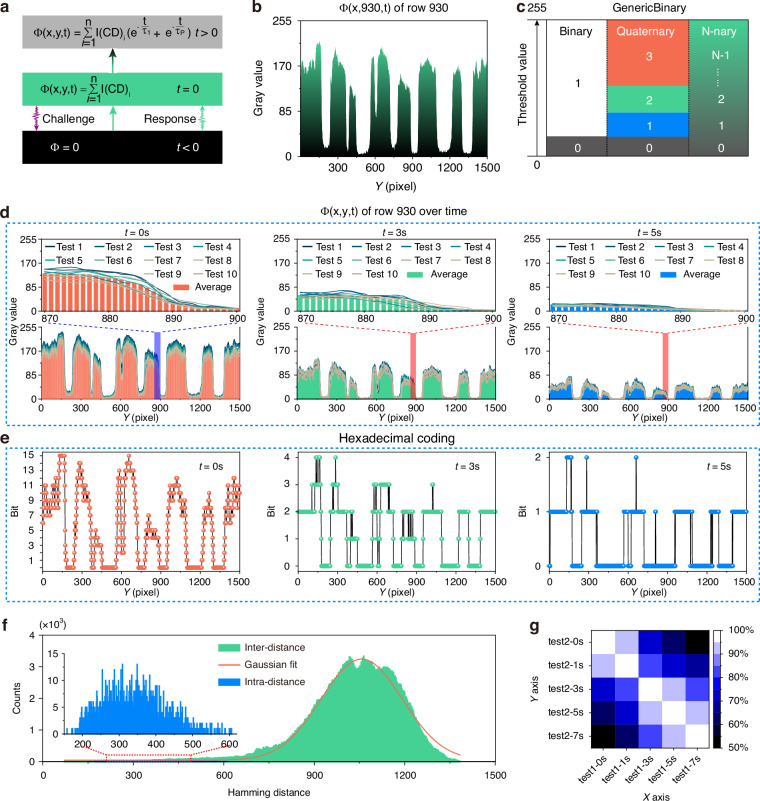
**N-nary TD-PUFs via time slice**. **a** Flow chart of the PMF ($\varPhi$) of the TD-PUFs before and after the excitation challenge of light. **b** The PMF ($\varPhi$) of the TD-PUFs along (x, 930) axes at t = 0 s. **c** The schematic diagram of from binary, quaternary, to N-nary coding by selecting appropriate thresholds. **d** The PMF (${\varPhi }_{(x,930,t)}$) of the TD-PUFs measured for 10 times under the same excitation conditions at t = 0 s, 3 s, and 5 s, respectively. **e** The corresponding hexadecimal coding of the PMF (${\varPhi }_{(x,930,t)}$) with average value from 10 measurements at t = 0 s, 3 s, and 5 s, respectively. **f** The inter-distance and intra-distance of the TD-PUFs with hexadecimal coding. **g** The TD-PUFs’ statistical similarity result after two repeated measurements at five different moments

Compared with conventional PUFs, TD-PUFs integrate the time dimension in addition to the spatial dimension^51^, presenting a time evolution feature. At time t < 0, the PMF ($\varPhi$) equals to zero; after challenge the PMF ($\varPhi$) exhibits a response with value of $\mathop{\sum }\nolimits_{i=1}^{n}{{\rm{I}}({\rm{CD}})}_{i}$ at *t* = 0; subsequently, the PMF ($\varPhi$) decays exponentially over time, as illustrated in Fig. 4a. To provide a clearer explanation of how the response of each pixel within a TD-PUF changes over time, we randomly selected one row of data from the 1500 × 1500 pixels TD-PUF for analysis, and the PMF ${\varPhi }_{(x,\mathrm{930,0})}$ is shown in Fig. 4b. Here, the intensity of the CDs within a pixel was transformed into gray value (the value was located in the range of from 0 to 255). The PMF ${\varPhi }_{(x,(y=\mathrm{50,200,500,800,1200}),0)}$ of the TD-PUFs are shown in Supplementary Fig. S20, and the statistical results clearly confirm randomly distributed value. Figure 4c depicts the process from generating gray value data to its transformation into N-nary coding. We employed a single threshold (30) to generate binary coding, three thresholds (30, 150, and 200) to produce quaternary coding and multiple thresholds to produce N-nary coding^42,47^. As time progresses, the quaternary coding evolves as the grayscale values decrease at the same thresholds. We repeated the response test of the TD-PUFs for 10 times under the same excitation challenge to avoid the accidental variations, the gray value results at t = 0 s, 3 s and 5 s are shown in Fig. 4d, exhibiting high reliability and consistency. Figure 4e displays the statistic results of the coding for hexadecimal coding at *t* = 0 s, 3 s and 5 s, respectively. The results indicate that the coding in hexadecimal format also evolve randomly over time, elucidating why the original PUFs can generate new PUFs. Finally, the safety characterization results for the hexadecimal coding TD-PUFs also confirm its exceptional properties, as presented in Fig. 4f. Figure 4g and Supplementary Fig. S21 are the similarity of the two repeated test results of the TD-PUFs under the same time interval change. The results show that the new PUFs derived from the TD-PUFs changes randomly over time. Finally, a comparison between the TD-PUF and representative optical PUFs reported in previous studies was conducted (see Supplementary Table S5). The results highlight the superior performance of the TD-PUF in key evaluation metrics.

### The application of TD-PUFs

**Fig. 5:**
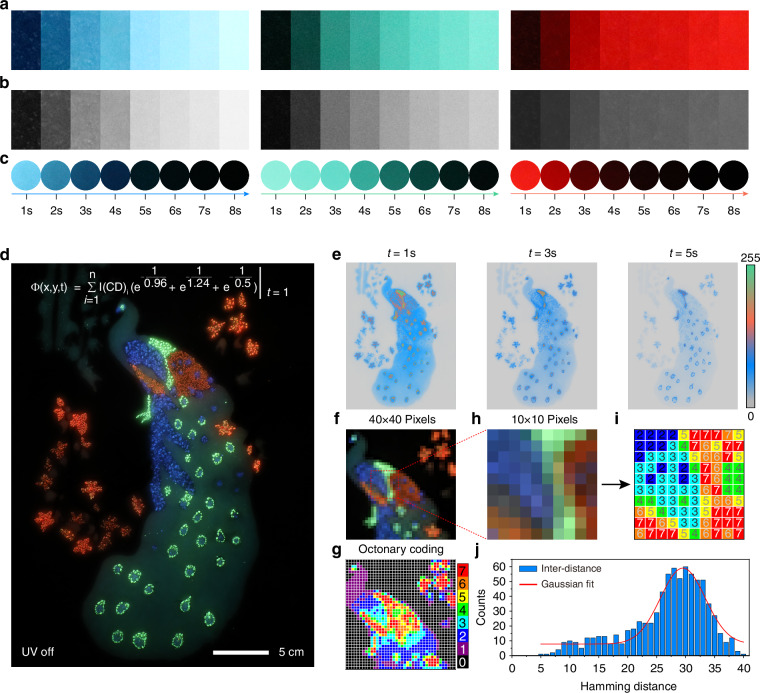
The application of TD-PUFs techology. **a** Phosphorescence images of films prepared with three types of CDs of different concentrations. **b** The grayscale images corresponding to phosphorescent images. **c** Phosphorescence photos of three CDs taken at different times. **d** Optical response photograph of the TD-PUFs painting at t = 1 s. **e** Pseudo-color images of the TD-PUFs painting at t = 1 s, 3 s, 5 s. **f** An optical photo of a 40 × 40 pixels area cut from the peacock painting. **g** Octonary coding map converted from the above image. **h** Magnified detailed optical photo of a 10 × 0 pixels area. **i** The corresponding octonary coding map of the peacock pattern in the 10 × 10 pixels area. **j** The Hamming intra-distance of the peacock pattern in the 40 × 40 pixels area

TD-PUFs exhibit large information storage capabilities due to the introduction of temporal dimension, making this technology well-suited for information storage and anti-counterfeiting^7,52,53^. Diverse anti-counterfeiting labels have been manufactured through the TD-PUFs technology, which can be affixed to the surfaces of significant and valuable objects^54,55^ (Supplementary Figs. S22 and S23). In addition, we further expanded the application of TD-PUFs technology to anti-counterfeiting painting, as shown in Fig. 5. We selected three CDs with different lifetimes and phosphorescence for the preparation of TD-PUFs painting. Supplementary Figs. S24 and S25 respectively demonstrate the optical stability of CDs at different temperatures, different humidities, and different excitation wavelengths to ensure the multi-scenario application capability of TD-PUFs in actual environments. The absorbance spectra, wavelength-dependent phosphorescence spectra, color coordinates, and photostability of the three CDs are shown in the Supplementary Fig. S26. It is worth noting that R-CDs utilize Förster resonance energy transfer (FRET) to achieve highly efficient red phosphorescence emission. In this system, Rhodamine B serves as the donor and the CDs act as the acceptor, allowing for efficient energy transfer. In order to demonstrate the effect of the CD content on their luminescence intensity, the three kinds of CDs were dispersed in polydimethylsiloxane (PDMS) to prepare films of different contents (see the method for the specific preparation process). Under the excitation of 365 nm light, the phosphorescence intensity of the three kinds of CDs increased with the increase of content. Figure 5a shows the phosphorescence photos of the films with different contents of CDs (the pictures were taken at t = 1 s) and converted them into grayscale images for analysis (Fig. 5b). Randomly draw a horizontal line on these grayscale images, and the average grayscale values along the line in each image are shown in the Supplementary Fig. S27. The increased grayscale values with the increase of CD contents in a unit volume indicate that they can be used for multi-level and vivid painting. In addition, they exhibit a time-dependent emission properties with emission lasting for more than 8 seconds, indicating their potential for TD-PUFs painting. A TD-PUFs peacock pattern painting was created through etching technology, incorporating three kinds of CDs with distinct triplet exciton emission colors and lifetimes, as illustrated in Supplementary Fig. S28. The phosphorescence spectra and decay curves of the three CDs are presented in Supplementary Figs. S29 and S30. The emission wavelength peaks are centered around 430 nm, 510 nm and 585 nm, with corresponding lifetimes of 0.63 s, 0.96 s, and 0.55 s, endowing the painting with vibrant color (Fig. 5c). The vibrant emission and diverse lifetime of the CDs endow the painting with a greater variety of changes, making it highly resistant to forgery. A 30 × 40 cm^2^ TD-PUFs peacock painting (*t* = 1 s) is presented in Fig. 5d and Supplementary Fig. S31, the painting produces a visually response over time, seemingly coming to life. The response value of each pixel of the pattern can be expressed using the following PMF:

10 $${\varPhi }_{(x,y,t)}=\mathop{\sum }\limits_{i=1}^{n}{I({\rm{CD}})}_{i}\left({e}^{\frac{-t}{0.96}}+{e}^{\frac{-t}{1.24}}+{e}^{\frac{-t}{0.5}}\right)$$

Where ${\varPhi }_{(x,y,t)}$ is the response value of the pixel at coordinates (x,y) at time t, ${{\rm{I}}({\rm{CD}})}_{i}$ is the intensity of the $i$-th CD within the pixel, ${e}^{\frac{-t}{0.96}}+{e}^{\frac{-t}{1.24}}+{e}^{\frac{-t}{0.5}}$ represent the exponential decay functions corresponding to the lifetimes of blue, green, and red emissions, respectively. Figure 5e shows the response value maps of the painting at *t* = 1 s, *t* = 3 s, and *t* = 5 s. A gradual weakening of intensity can be observed across different regions of the peacock’s body over time, exhibiting unpredictability until eventually stabilizing. The progression illustrates how the painting’s response changes over time, with varying grayscale values indicating different levels of luminescence due to the different contents and decay lifetimes. Furthermore, a section of the painting was tailored to a 40 × 40 pixels image for coding analysis, as shown in Fig. 5f, and then it was converted into octonary coding, as shown in Fig. 5g. Figure 5h magnifies a smaller section (10 × 10 pixels) from the previous 40 × 40 pixels area, and the corresponding detailed octonary coding matrix for the 10 × 10 pixels magnified image is shown in Fig. 5i. Notably, the inter-distance results presented in Fig. 5j indicate that the TD-PUFs painting demonstrates robust internal randomness. The vibrant triplet exciton emissions and diverse lifetimes of the CDs result in dynamic and visually striking changes in the painting over time, making it highly resistant to forgery.

## Discussion

In summary, TD-PUFs technology has been proposed and demonstrated in this work, and the introduction of temporal dimension endow it with a dynamic and versatile capability for anti-counterfeiting, information storage, and visual representation. The CD with long-lived and bright triplet excitons serves as the cornerstone for the construction of TD-PUFs, breaking the spatial limitation and increasing the difficulty of being attacked. Moreover, we have built PMF by resolving boundary conditions for description the temporal evolution of TD-PUF with time. Furthermore, we showcased the versatility of TD-PUFs through flexible labeling and highlighting their anti-counterfeiting for valuable items. In the end, TD-PUFs painting has been demonstrated, the vibrant emission and diverse lifetime of the CDs endow the panting with a greater variety of changes, making it highly resistant to forgery. The introduction of TD-PUFs technology in this study signifies a departure from the spatial limitations inherent in traditional PUFs, thus paving the way for significant advancements in PUF development within the temporal dimension.

## Materials and methods

### Materials

The precursors used in this study include poly acrylic acid ([C_3_H_4_O_2_]_n_, purity > 99.5%), 1,3-diamino propane (C_3_H_10_N_2_, purity > 98%), Ethylenediamine (EDA, purity > 99%), phosphoricacid (purity > 95%), rhodamine B (C_28_H_31_ClN_2_O_3_, purity > 99%), biuret (C_2_H_5_N_3_O_2_, purity > 98%), silicic acid (C_8_H_20_O_4_Si, Purity > 98%), ammonium hydroxide solution (H_5_NO, purity > 98%). All the chemicals were purchased from Macklin Chemistry Co. Ltd (Shanghai, China). Note that all the chemicals used in this work were analytical grade without further purification.

### Synthesis of blue phosphorescence CDs (B-CDs)

Firstly, 0.45 g of poly (acrylic acid) powders were dissolved in 20 mL of deionized water. Then 0.85 mL of 1,3-diaminopropane was added to the poly (acrylic acid) aqueous solution with stirring for 5 min. Then, the resulting solution was transferred to a 50 ml Teflon-lined stainless-steel autoclave. The sealed autoclave was heated to 200 °C and maintained at this temperature for 8 h. Afterward, the reactor was cooled naturally to room temperature. The aqueous solution was then centrifuged for 10 min to remove sediment. The supernatant was filtered through a 0.22 μm membrane, and the resulting filtrate was collected. Next, 1 mL of the supernatant aqueous solution and 0.5 mL of tetraethoxysilane (TEOs) were dispersed in 15 mL of deionized water to form a solution. Then, 0.5 mL of ammonia was added to the solution and stirred at room temperature for 8 h. Finally, the blue phosphorescent CNDs can be obtained by drying at 60 °C.

### Synthesis of green phosphorescence CDs (G-CDs)

1.0 g of biuret precursors were mixed in a mortar and ground uniformly for 10 min. The resulting solid mixture was removed to a Teflon-lined stainless-steel autoclave and heated at 190 °C for 10 h. The resulting CNDs sample was allowed allowed to cool slowly to room temperature.

### Synthesis of red phosphorescence CDs (R-CDs)

First, 1.0 mL of ethylenediamine (EDA) solution was dissolved in 15 mL of deionized water. Next, 2 mL of phosphoric acid was added to the EDA aqueous solution and stirred for 5 min. The resulting transparent solution was heated in a microwave oven (750 W) for 120 s. After cooling room temperature, 20 mL of deionized water was added, resulting in a light-yellow solution. The solution was then centrifuged for 10 min to remove sediment. The resulting supernatant was collected. 1 mL of the CNDs supernatant aqueous solution, 1 mL of Rhodamine B (4.5 mg/mL), and 1 mL of tetraethoxysilane (TEOs) were mixed in 16 mL of deionized water to form a solution. Then, 1 mL of ammonia was added, and stirred at room temperature for 8 h. Finally, the red phosphorescent CNDs were obtained by drying at 60 °C.

### Fabrication and readout of TD-PUFs

First, CD particles of various sizes were randomly selected and scattered on the black background. Next, the particles were stimulated using 365 nm ultraviolet light in a dark environment. After removing the ultraviolet light source, a camera was used to capture phosphorescence response images at different time intervals. These optical images were converted into corresponding binary codes, which serve as the fundamental elements of TD-PUF. All PUFs exhibiting temporal changes over time constitute TD-PUFs.

### Preparation of PDMS film based on B-CDs

Eight B-CD samples, with masses in an arithmetic progression ranging from 0.02 g to 0.32 g, (with a 0.04 g interval), were accurately weighed. Each sample was mixed with 2 g of PDMS. The mixtures were subsequently placed in a drying oven and heated at 60 °C for 2 h. After heating, the PDMS films embedded with CDs were exposed to a 36 nm ultraviolet lamp. After ceasing of the UV excitation, phosphorescence images of the films were captured using a camera. The resultant phosphorescence images exhibited varying luminescence intensities corresponding to the different masses of CDs in the PDMS films.

### Preparation of PDMS film based on G-CDs and R-CDs

The preparation methods for PDMS films incorporating G-CDs and R-CDs are identical to those used for B-CDs.

### Digitization of PUFs

To generate binary coding or N-nary coding from PUF images, MATLAB was used to design a specialized program for image processing and digital key extraction. The main processing steps are as follows:

1. *Converting images to grayscale images*: Photos taken with a camera or mobile phone were imported into MATLAB. The photos are then converted into corresponding grayscale images.
2. *Digitizing the grayscale image into base on N-nary coding*: A suitable threshold within the digital range of 0-255 was selected to convert the grayscale image into binary coding or N-nary coding.
3. *Calculating the Hamming distance*: For binary coding, two sets of PUF data were imported into the MATLAB to generate the corresponding data matrices. Appropriate thresholds were chosen to form a binary matrix, with values above and below the threshold defined as “1” (white) and “0” (black), respectively. The inter-distance was determined by calculating the Hamming distance within each PUF, while the intra-distance was measured by calculating the Hamming distance between different PUFs. The similarity index is computed as an intuitive performance metric between two PUFs, based on the statistical result of the Hamming distance. The percentage of differing pixels (represented as “0”) relative to the total number of pixels was used to determine the similarity.

## Supplementary information

Supplementary information

## Data Availability

The data that support the findings of this study are available from the corresponding author upon request. Source data are provided with this paper.
