# Supplementary material for: Time-dependent physical unclonable functions by long-lived triplet excitons in carbon dots
Source: Light Sci Appl. 2025 Aug 20;14:283. doi: 10.1038/s41377-025-01940-9 (PMC12368053; doi:10.1038/s41377-025-01940-9)
Supplement: Supplementary file 1 — Supplementary information [file 41377_2025_1940_MOESM1_ESM.docx]

**Supplementary information**

**Time-Dependent Physical Unclonable Functions by Long-Lived Triplet Excitons in Carbon Dots**

Yan-Wei Hu^1^, Qing Cao^1^, Shi-Yu Song^1^, Yuan Sun^1^, Ya-Chuan Liang^2^, Wen-Bo Zhao^1^, Chao-Fan Lv^1^, Chong-Xin Shan^1✉^, Kai-Kai Liu^1,3✉^

^1^Henan Key Laboratory of Diamond Optoelectronic Materials and Devices, School of Physics and Laboratory of Zhongyuan Light, Zhengzhou University, Zhengzhou, China.

^2^School of Electronics and Information, Zhengzhou University of Light Industry, Zhengzhou, China.

^3^Institute of Quantum Materials and Physics Henan Academy of Sciences Zhengzhou 450046, China

^✉^e-mail: cxshan@zzu.edu.cn; [liukaikai@zzu.edu.cn](mailto:liukaikai@zzu.edu.cn).

**Table of contents:**

**Supplementary Fig. S1** Synthesis schematic diagram of TD-PUFs based on CDs.

**Supplementary Fig. S2** Optical photos and particle size statistics of CDs of three different sizes.

**Supplementary Fig. S3** A schematic diagram illustrating the evolution of the TD-PUFs over time.

**Supplementary Fig. S4 and Fig. S5** Optical and structure characterization of CDs

**Supplementary Fig. S6** Phosphorescence images of TD-PUFs changing over time after excitation.

**Supplementary Fig. S7** Phosphorescence intensity and lifetime of CDs measured across a temperature range from –100 °C to +70 °C.

**Supplementary Fig. S8** Photoluminescence (PL) spectra, phosphorescence intensity spectra, and phosphorescence lifetime of CDs measured under different relative humidity levels (11% to 84%).

**Supplementary Fig. S9** PL spectra and phosphorescence intensity spectra of CDs measured under different atmospheric conditions (air, argon, nitrogen, oxygen, and hydrogen).

**Supplementary Fig. S10** PL spectra and phosphorescence intensity spectra of CDs measured after different storage durations (0, 30, and 180 days).

**Supplementary Fig. S11** The changes in binary and quaternary coding of TD-PUFs over time.

**Supplementary Fig. S12** The Hamming inter-distance of the TD-PUF obtained by Standard-HD and Fractional-HD.

**Supplementary Fig. S13 and Fig. S14** Three sizes of CD were used to prepare TD-PUFs.

**Supplementary Fig. S15** The Hamming inter-distance of the TD-PUF_1_ by measured at t = 2 s.

**Supplementary Fig. S16** Hamming intra-distance statistical image of binary coding that measuring the same PUF twice at four moments.

**Supplementary Fig. S17** Hamming intra-distance statistical image of quaternary coding that measuring the same PUF twice at four moments.

**Supplementary Fig. S18** Hamming intra-distance statistical maps of binary coding, where eight PUFs were measured twice at the same moment (t = 0.5 s).

**Supplementary Fig. S19** Similarity statistical image of the TD-PUF with binary coding measured twice at four different moments.

**Supplementary Fig. S20** The corresponding PMF ($\Phi$) of the TD-PUFs along (x, 50), (x, 200), (x, 500), (x, 800), (x, 1200) axes at t = 0 s.

**Supplementary Fig. S21** The TD-PUFs’ similarity result after two repeated measurements at three different moments.

**Supplementary Fig. S22** Practical applications of TD-PUFs.

**Supplementary Fig. S23** Uniqueness and repeatability of the PUF label affixed to medicine bottle.

**Supplementary Fig. S24** Optical stability of TD-PUF at 15°C and 40°C.

**Supplementary Fig. S25** Optical stability of the three CDs at different humidities and different excitation wavelengths.

**Supplementary Fig. S26** The absorbance spectra, wavelength-dependent phosphorescence spectra, color coordinates, and photostability of the three CDs.

**Supplementary Fig. S27** Grayscale images corresponding to the color scale images prepared using three different concentrations of CDs, along with the statistical results of the corresponding grayscale values.

**Supplementary Fig. S28** Flow chart of peacock pattern preparation and an optical photograph taken in sunlight.

**Supplementary Fig. S29** Phosphorescence spectra of three kinds of CDs.

**Supplementary Fig. S30** Phosphorescence lifetime curves of three kinds of CDs.

**Supplementary Fig. S31** Photos of TD-PUFs based on a carved peacock painting.

**Supplementary Table S1** Encoding capability of different pixel TD-PUFs.

**Supplementary Table S2** Uniformity of different TD-PUFs.

**Supplementary Table S3** The similarity index of PUFs with different pixels.

**Supplementary Table S4** The similarity index of two patterns’ PUF over time.

**Supplementary Table S5** Supplementary Table 5. Performance comparison between the TD-PUF and representative optical PUFs reported in previous studies.

During the synthesis process, carbon dots (CDs) are assembled into particles of various shapes and sizes, with their positions on the substrate being unpredictable. This ensures that the prepared TD-PUFs possess irreproducible capabilities. However, to obtain TD-PUFs with uniform size, carbon dot particles can be screened using a sieve (Supplementary Fig. S1a). The UV lamp generates 365 nm excitation light to challenge the TD-PUFs, while the camera captures the optical images produced in response. Subsequently, these evolving PUFs are digitally processed to generate binary codes, which serve as the foundational keys for information encryption (Supplementary Fig. S1b).

**
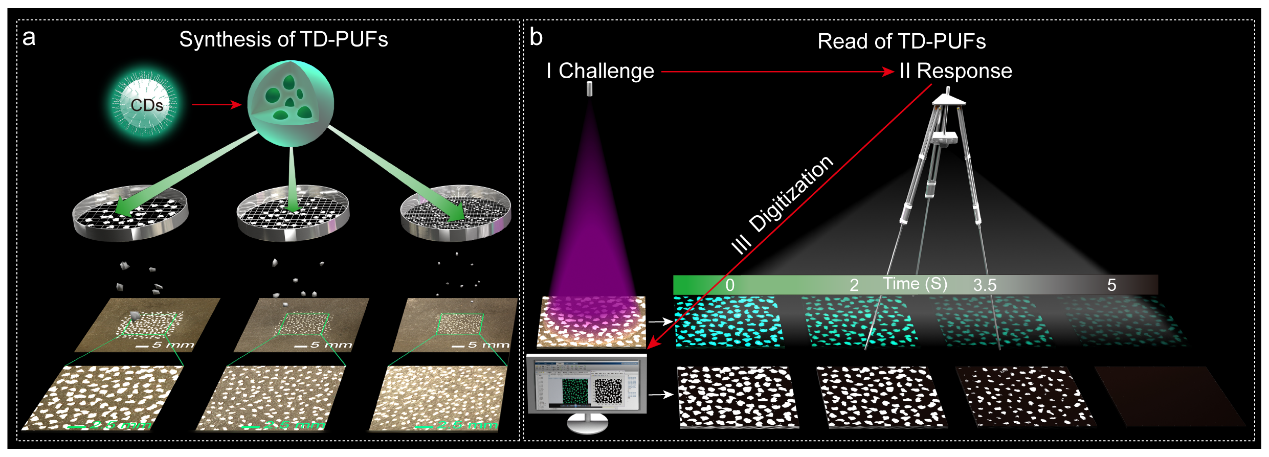
**

**Supplementary Fig. S1 a** The preparation process of TD-PUFs. **b** Operation process of TD-PUFs.


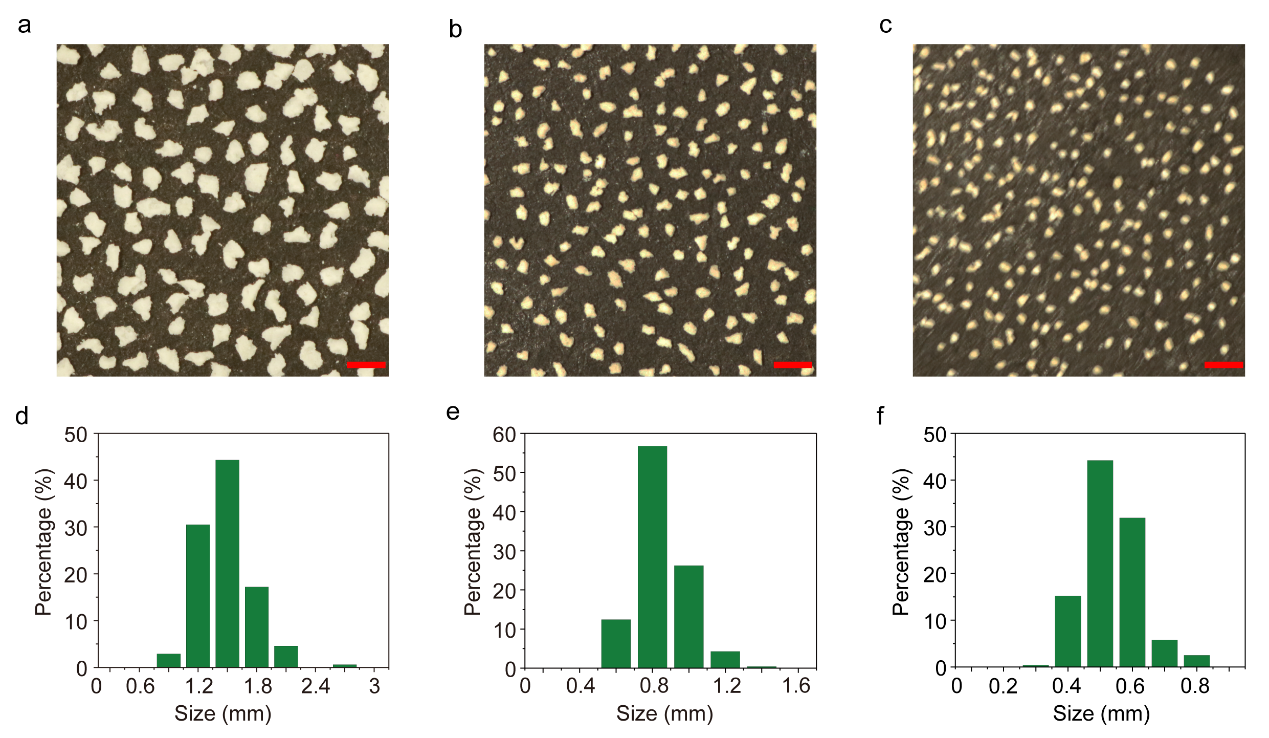


**Supplementary Fig. S2 a**, **b**, **c** Optical images of TD-PUFs prepared using carbon dot particles of three different sizes. **d**, **e**, **f** Statistical results of the average particle size for carbon dot particles in three TD-PUFs. All scale bars represent 2 mm.


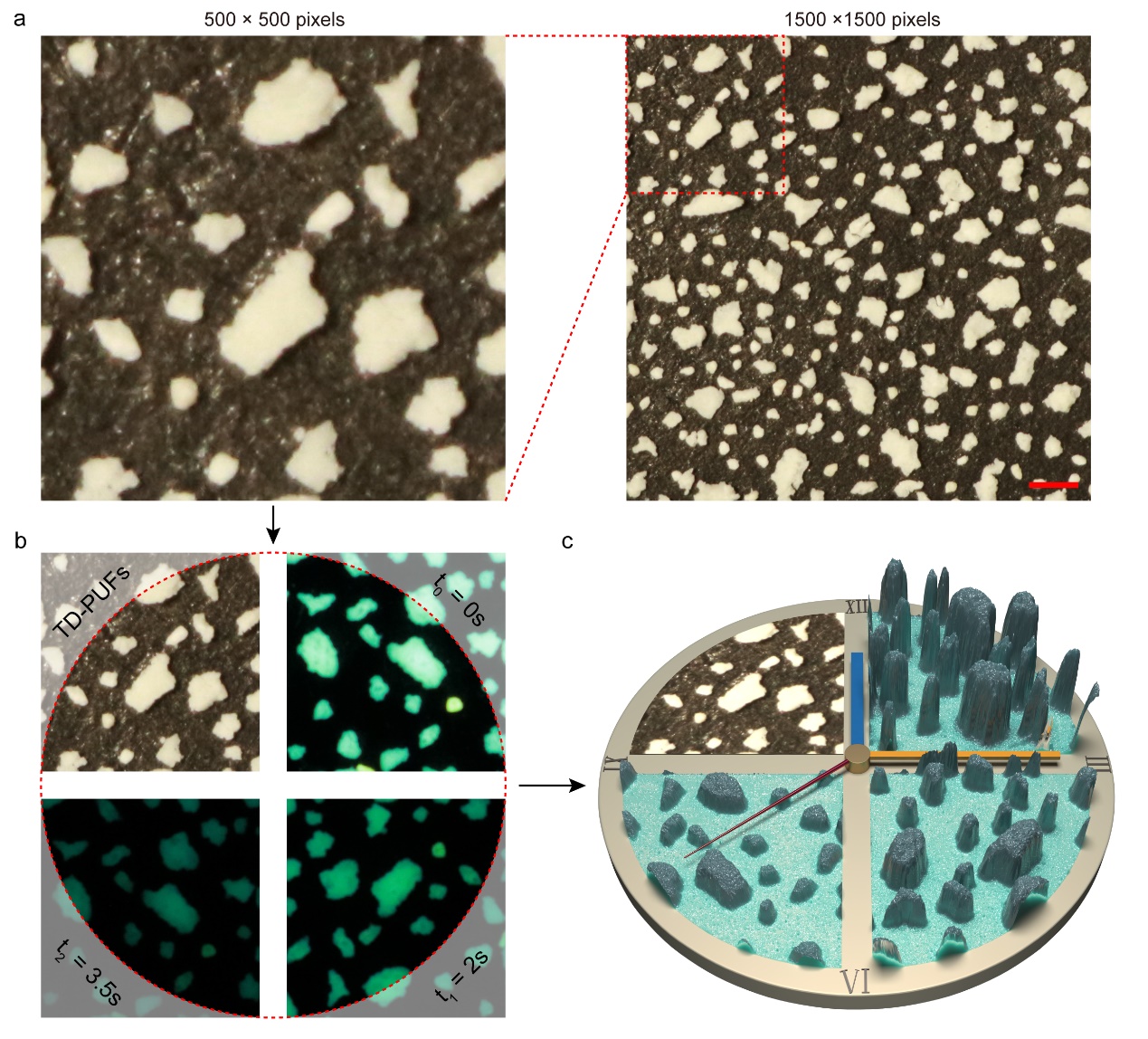


**Supplementary Fig. S3 a** Optical image (1500 × 1500 pixels) of TD-PUFs prepared by mixing carbon dot particles with different morphologies and sizes, with a section of the image (500 × 500 pixels) extracted for analysis. **b** Natural light photos of TD-PUFs and their optical response images after UV light excitation are presented in 90-degree fan structures, along with **(c)** the corresponding three-dimensional graphics (c). The scale bar represents 2 mm.

**Characterization of CDs**

The CDs are synthesized in a single step using solid-state heating. Subsequently, their morphology and structure are characterized. The TEM images of CDs, shown in Supplementary Fig. S4a, reveal nanoparticles with a particle size uniformly distributed around 4 nm. The inset image highlights the crystal properties, with a lattice spacing of 0.21 nm. The FTIR spectra of the CDs, shown in Supplementary Fig. S4b, exhibit stretching vibrations corresponding to O-H at 3472 cm⁻¹ and C=O at 1541 cm⁻¹. Moreover, compared to the precursors, the low-field shift of the C=O stretching vibration and the broadening of the -OH peak suggest the formation of hydrogen bonds in the CDs. Moreover, compared to the precursors, the low-field shift of the C=O stretching vibration and the broadening of the -OH peak suggest the formation of hydrogen bonds in the CDs. Thermo-gravimetric analysis (TGA) of CDs, as shown in Supplementary Fig. S4c, was performed to provide a detailed explanation of the formation of hydrogen bonds. The survey X-ray photoelectron spectroscopy (XPS) spectra of the CDs, presented in Supplementary Fig. S5, reveal the formation of chemical bonds. Consequently, the above analyses reveal the cause of phosphorescence in CDs and propose a mechanism involving hydrogen bond restriction that enhances phosphorescence emission, as illustrated in Supplementary Fig. S4d. The oxygen atom, with its higher electronegativity and smaller atomic radius, facilitates the formation of hydrogen bonds between the C=O group and water molecules. The formation of intermolecular hydrogen bonds restricts the rotation and vibration of the molecules, increases the rigidity of the entire system, and leads to enhanced phosphorescence. For convenience, the carbon nanodots are photographed and displayed. Supplementary Fig. S4e shows images of the nanodots with ultraviolet light turned on and off.

The photophysical properties of the CDs are investigated, as shown in Supplementary Fig. S4f-k. In the powder state, the fluorescence and phosphorescence emission peaks of the CDs are located at 405 nm and 510 nm, respectively (Supplementary Fig. S4f). The low-temperature fluorescence and phosphorescence spectra of the CDs at 77 K also exhibit emission peaks at 405 nm and 510 nm, respectively (Supplementary Fig. S4g). Time-resolved fluorescence spectra were measured to evaluate the decay lifetime of the CDs in their emission regions, revealing a carrier lifetime of approximately 1.3 s (Supplementary Fig. S4h). Additionally, ultraviolet-visible absorption spectra demonstrate that CDs exhibit similar absorption profiles across different polar solvents, with a red shift in the absorption band at 210-240 nm, indicating n-π* transitions as solvent polarity increases (Supplementary Fig. S4j). Moreover, the CD powder shows a prominent peak in the wavelength range of 310 to 380 nm, reflecting a strong π-π* transition (Supplementary Fig. S5a). A proposed transition model is illustrated in Supplementary Fig. S4k: Oxygen atoms with lone pair electrons facilitate the transition of electrons from the singlet state to the triplet state. The strong hydrogen bonding between luminescent units of the CDs stabilizes the triplet electrons, effectively populating the triplet state. This is followed by the return of electrons to the S₀ state from T₁ with subsequent phosphorescence emission.


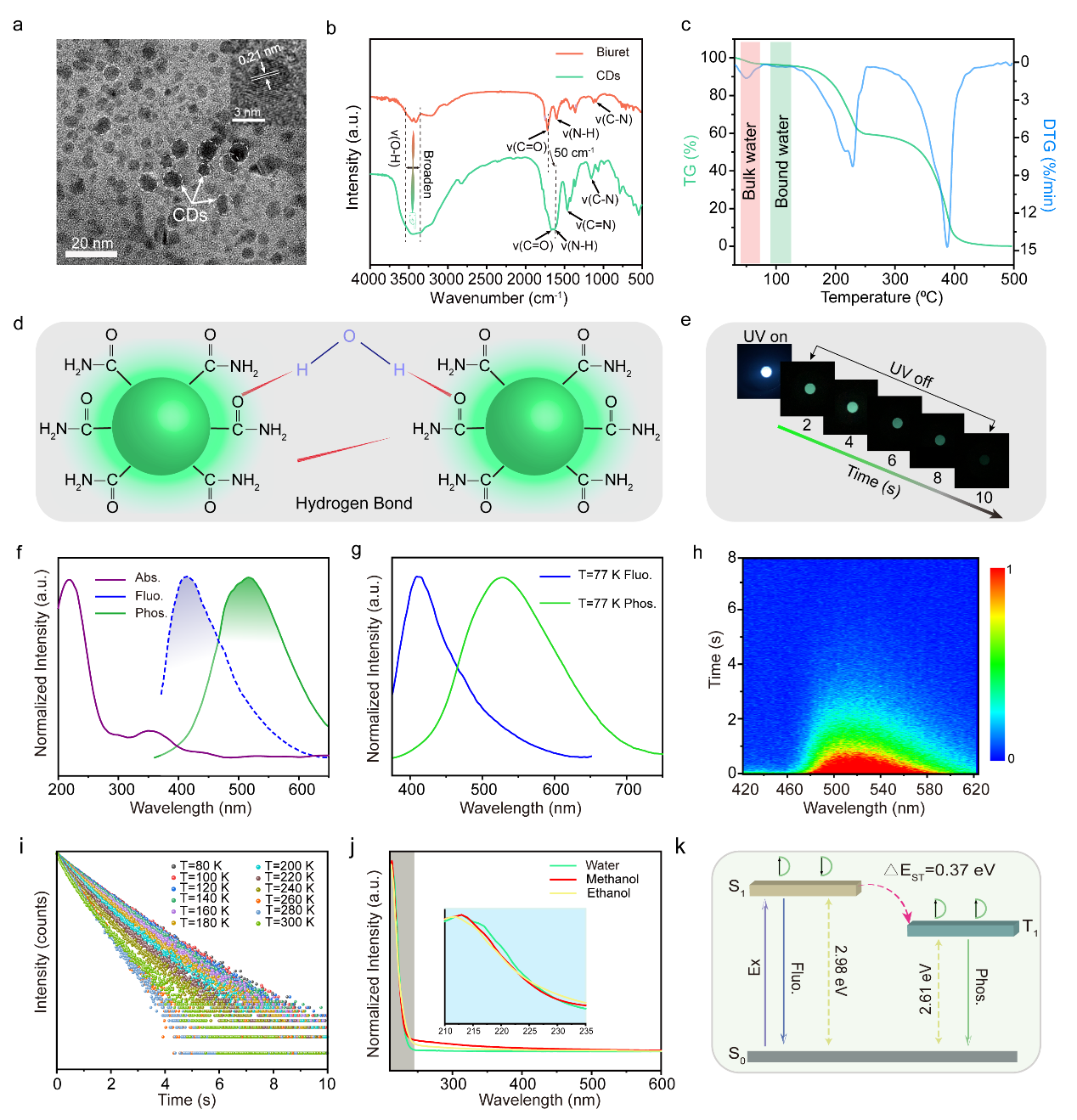


**Supplementary Fig. S4 a** TEM images of CDs. **b** FTIR spectra of CDs. **c** TGA curves of the CDs. **d** Schematic illustration of the molecular interactions between the CDs and water molecules. **e** Images of the CDs in UV on and UV off. **f** The absorption spectrum, normalized fluorescence spectrum and normalized phosphorescence spectrum of the sample were prepared. **g** Deconvolution of the low temperature (77 K) fluorescence (blue curve) and phosphorescence (green curve) spectra of CDs under excitation at 365 nm. **h** Time-resolved afterglow contour of CDs. **i** Temperature-dependent transient decay curves of the CDs. **j** UV–vis absorption spectra of the CDs in different polar solvents. **k** Proposed mechanism for

ultralong phosphorescence of CDs.


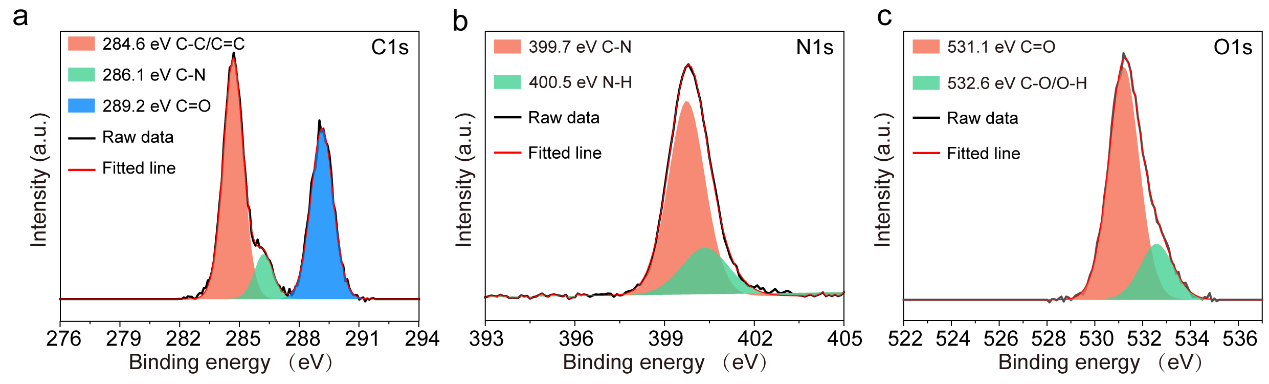


**Supplementary Fig. S5** C 1s **a** N 1s **b** and O 1s **c** spectra of the CDs.


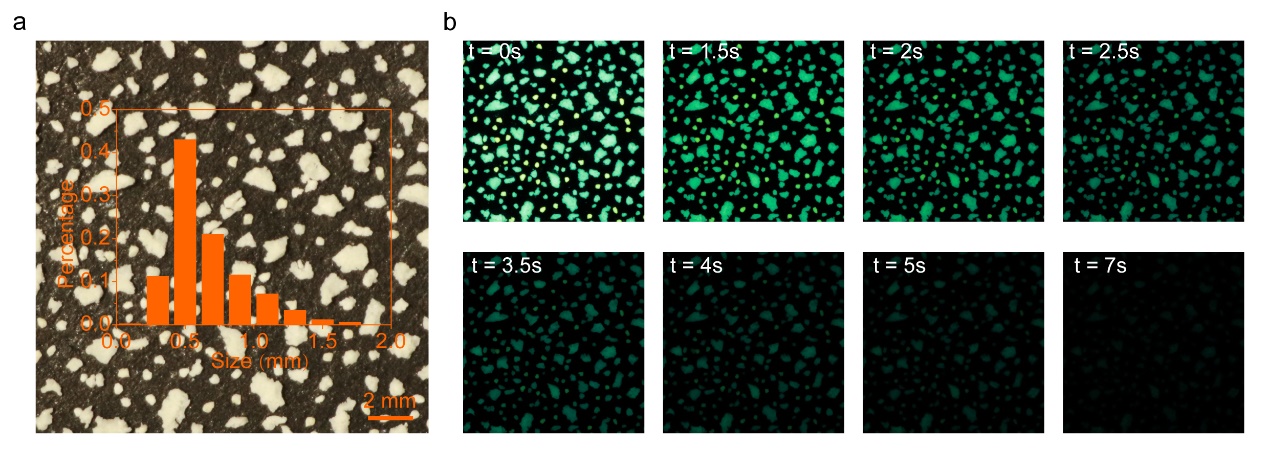


**Supplementary Fig. S6 a** Optical photos of TD-PUFs prepared from CDs with different luminescence intensity and sizes. **b** Phosphorescent photos of this TD-PUFs at different decay times.


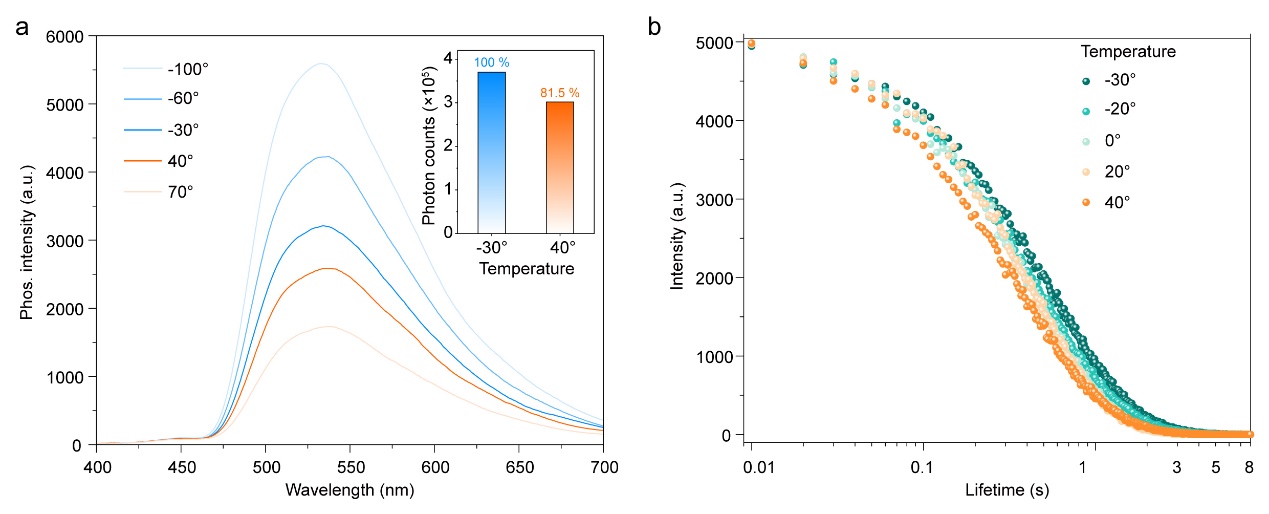


**Supplementary Fig. S7 a** Phosphorescence spectra of the CDs measured at different temperatures (−100 °C, −60 °C, −30 °C, +40 °C, and +70 °C). The inset shows the integrated emission areas at −30 °C and +40 °C. **b** Phosphorescence decay curves recorded at −30 °C, −20 °C, 0 °C, +20 °C, and +40 °C.


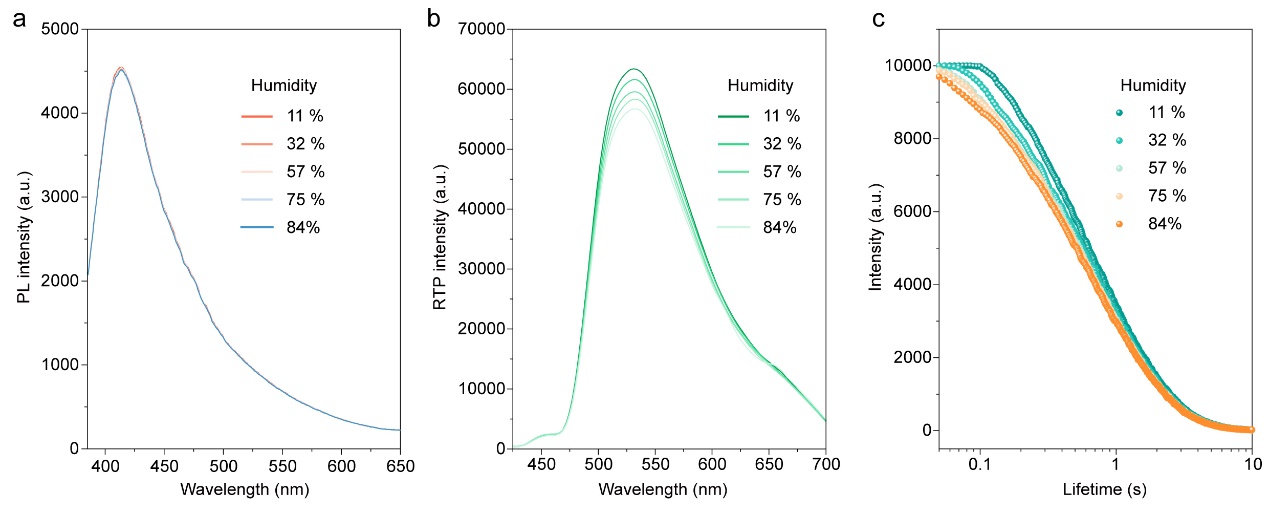


**Supplementary Fig. S8** **a** PL spectra of the CDs measured at relative humidity levels of 11%, 32%, 57%, 75%, and 84%. **b** Phosphorescence spectra of the CDs measured under the different humidity conditions. **c** Time-resolved phosphorescence decay curves recorded at each corresponding humidity level.


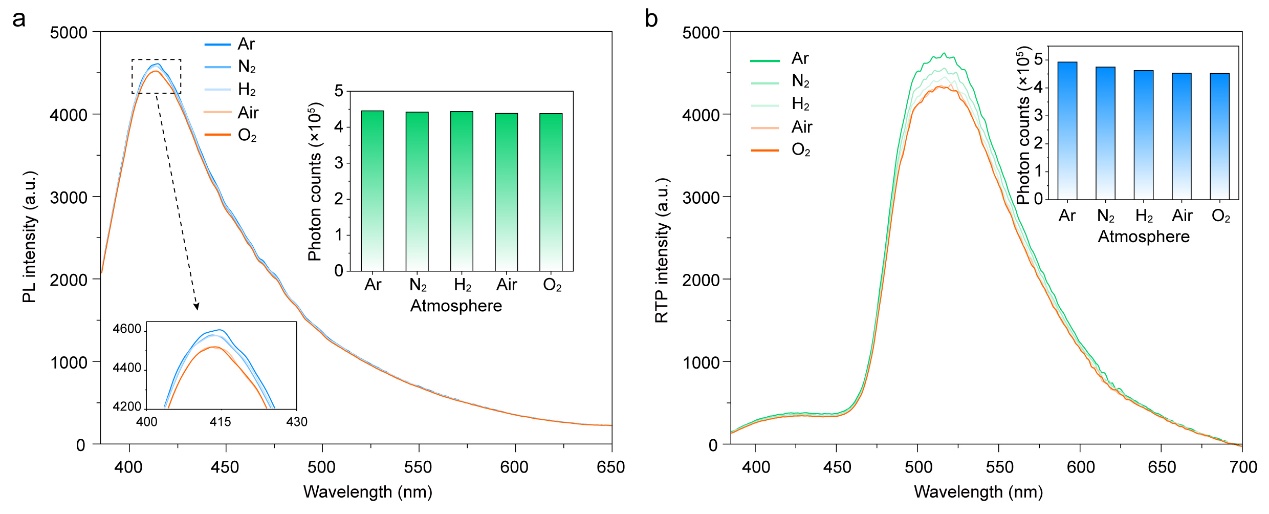


**Supplementary Fig. S9 a** PL spectra of the CDs under different atmospheric environments: Ar, N₂, H₂, air, and O₂. Inset shows the integrated PL area under each condition. **b** Phosphorescence spectra of the CDs under the same gas environments. Inset shows the corresponding integrated phosphorescence intensity.


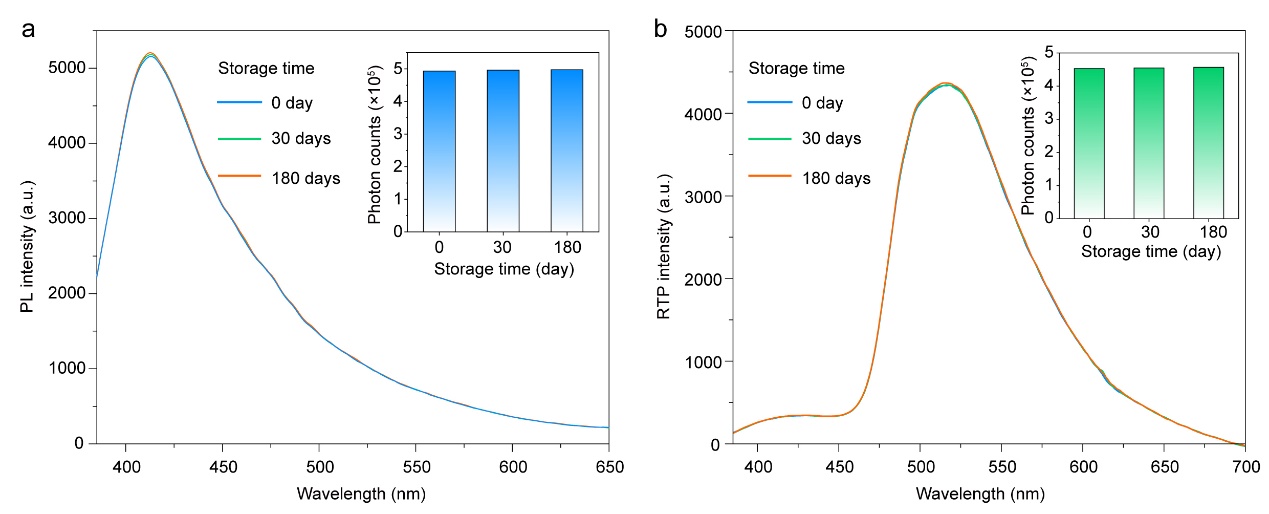


**Supplementary Fig. S10 a** PL spectra of the CDs after storage for 0, 30, and 180 days. Inset shows the integrated PL intensity for each time point. **b** Phosphorescence spectra of the CDs after storage for 0, 30, and 180 days. Inset shows the integrated phosphorescence intensity for each time point.

**
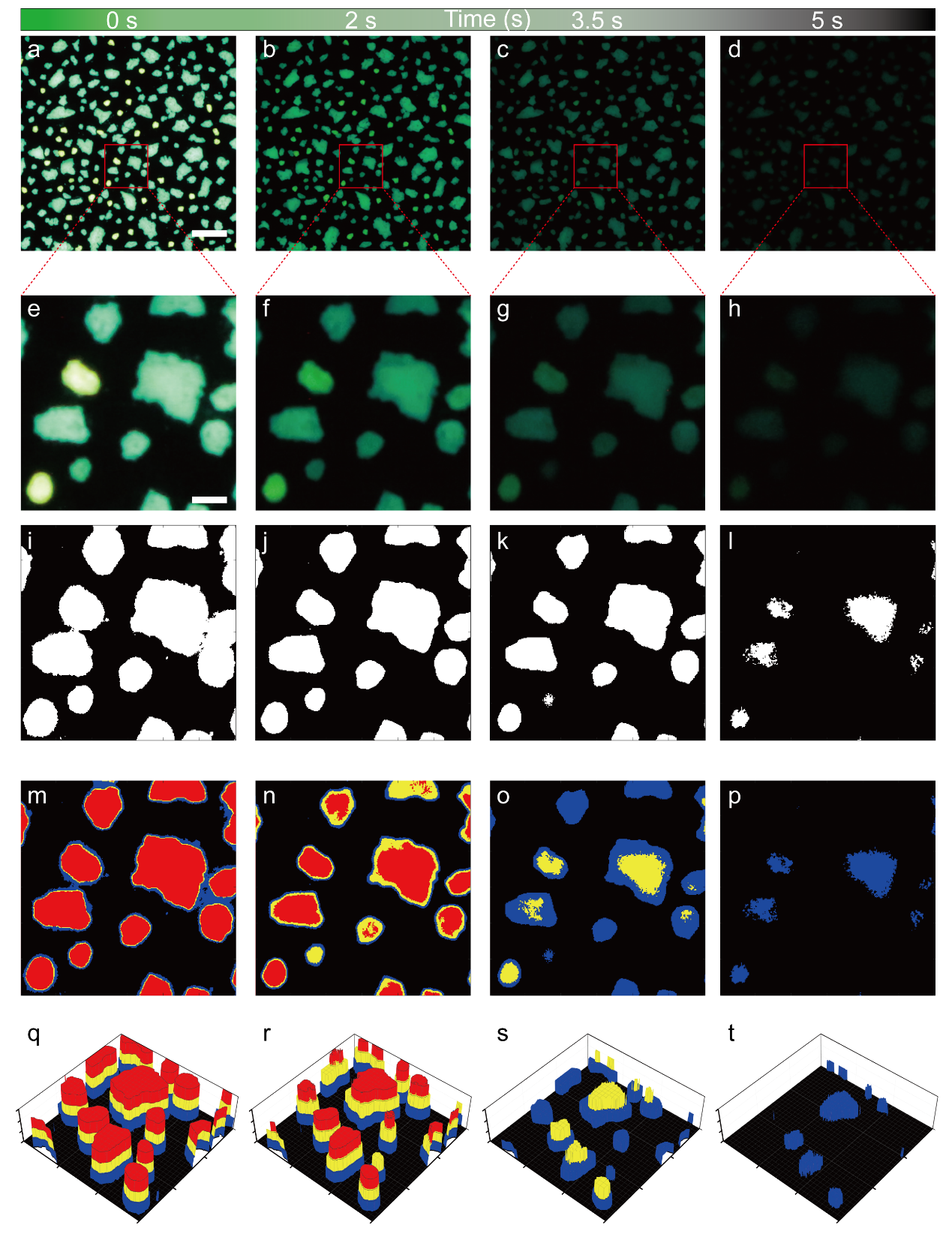
**

**Supplementary Fig. S11 a-d** Phosphorescence images of four moments in the response range (the scale bar is 2.5 mm), **e-h** their local area enlarged images (the scale bar is 500 μm). **i-l** The corresponding binary coding. **m-p** The corresponding quaternary coding. **q-t** The three-dimensional images of quaternary coding.


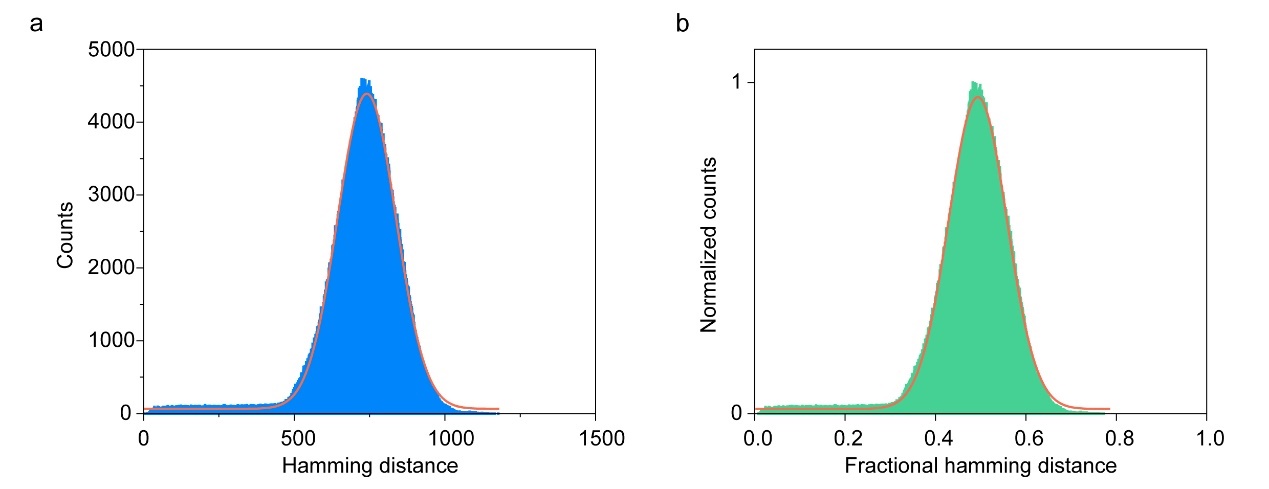


**Supplementary Fig. S12 a** The Hamming inter-distance of the TD-PUF obtained by Standard-HD. **b** The Hamming inter-distance of the TD-PUF obtained by Fractional-HD.

**Three sizes of CD were used to prepare TD-PUFs.**

Supplementary Fig. S9a-c illustrate the temporal changes in PUF images with dimensions of 1500 × 1500 pixels. The corresponding digitization results for binary and quaternary encoding are shown in Supplementary Fig. S9d-f and Fig. S9g-i, respectively. In binary encoding, '1' and '0' bits represent 'on' and 'off', respectively, whereas in quaternary encoding, '1', '2', and '3' denote 'on', and '0' represents 'off'. As depicted, '1' bits (white) in the binary encoding gradually transition to '0' bits (black) over time. This transition is attributed to variations in the number of CDs per pixel and their relative spatial positions, resulting in differing degrees of intensity change over time. Additionally, Supplementary Fig. S10 displays the time-dependent changes in PUFs with varying pixel dimensions (10 × 10, 100 × 100, 1000 × 1000). An increase in pixel number leads to a more complex response, making the time-dependent trends in PUFs more pronounced with higher pixel dimensions.


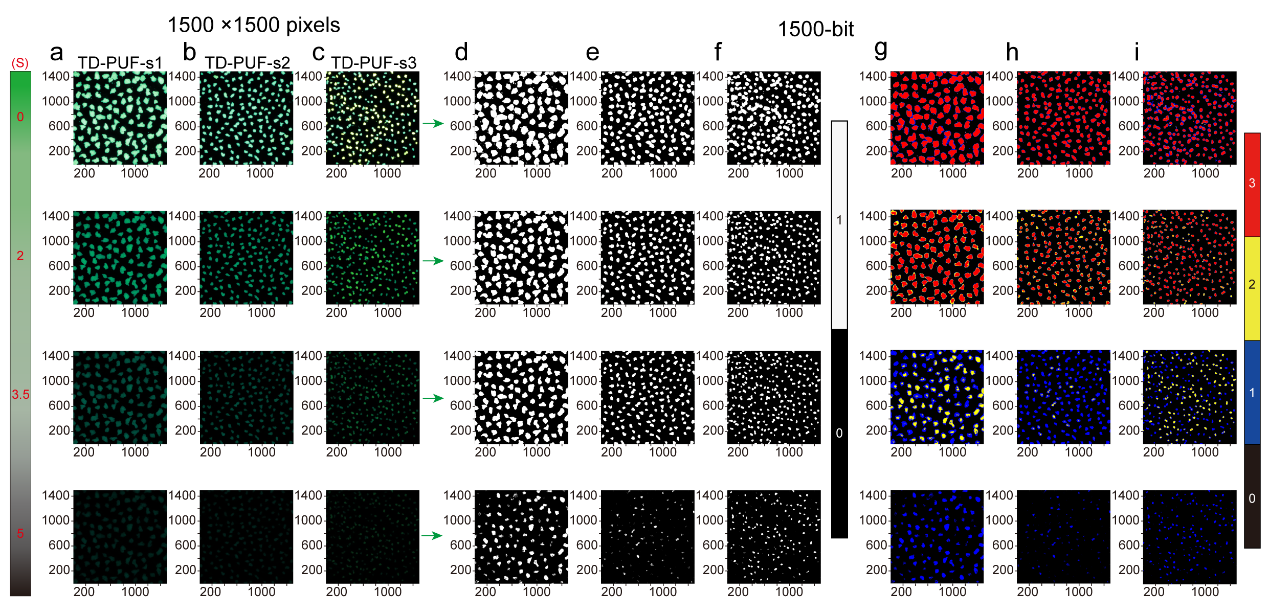


**Supplementary Fig. S13 a**-**c** The phosphorescent response photos of three sizes’ PUFs at eight moments respectively. **d**-**f** Corresponding maps of the binary coding. **g**-**i** Corresponding maps of the quaternary coding.


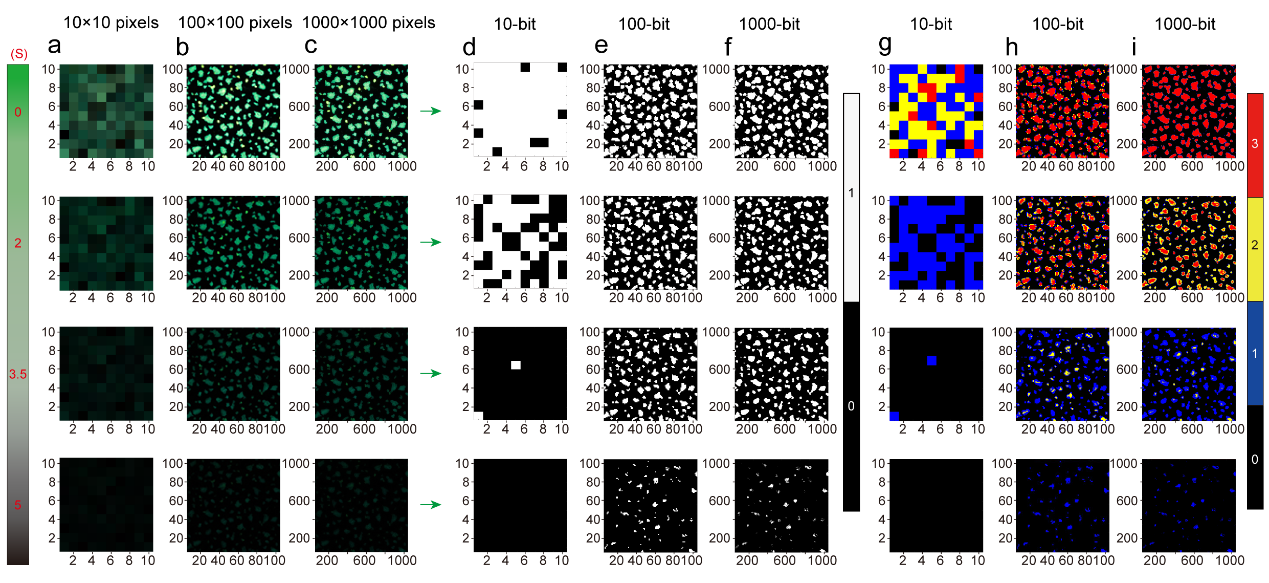


**Supplementary Fig. S14** The phosphorescent response photos of PUFs with 10$\times$10 pixels (**a**), 100$\times$100 pixels (**b**) and 1000$\times$1000 pixels (**c**) at eight moments respectively. **d-f** Corresponding atlas of the binary coding. **g-i** Corresponding atlas of the quaternary coding.


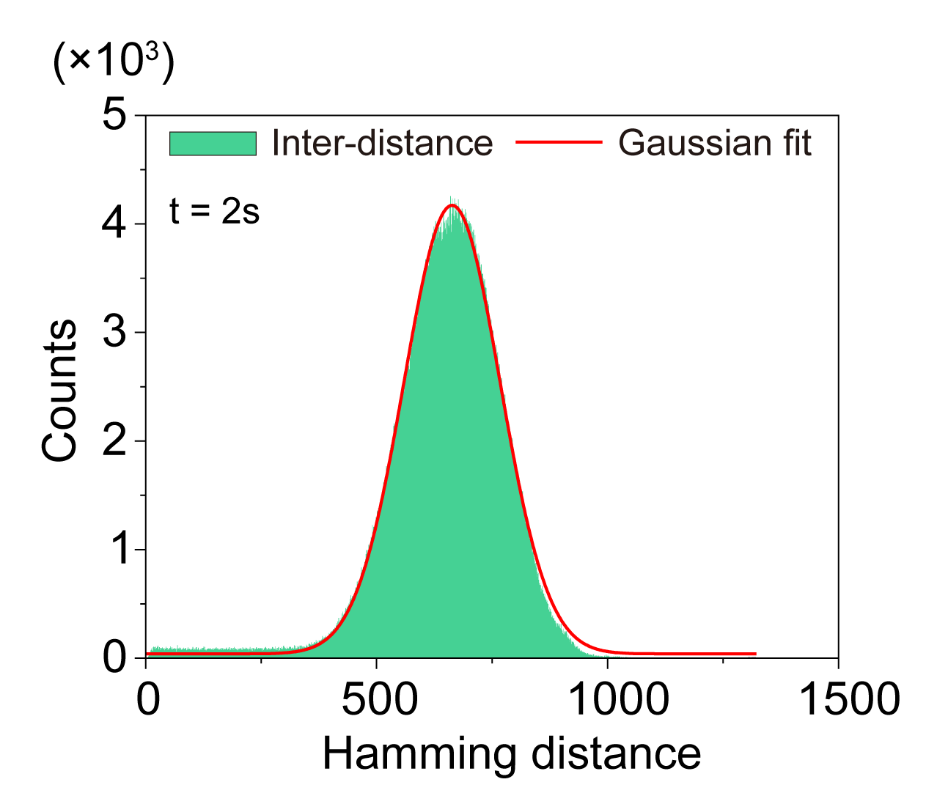


**Supplementary Fig. S15** The Hamming inter-distance of the TD-PUF_1_ by measured at t = 2 s.


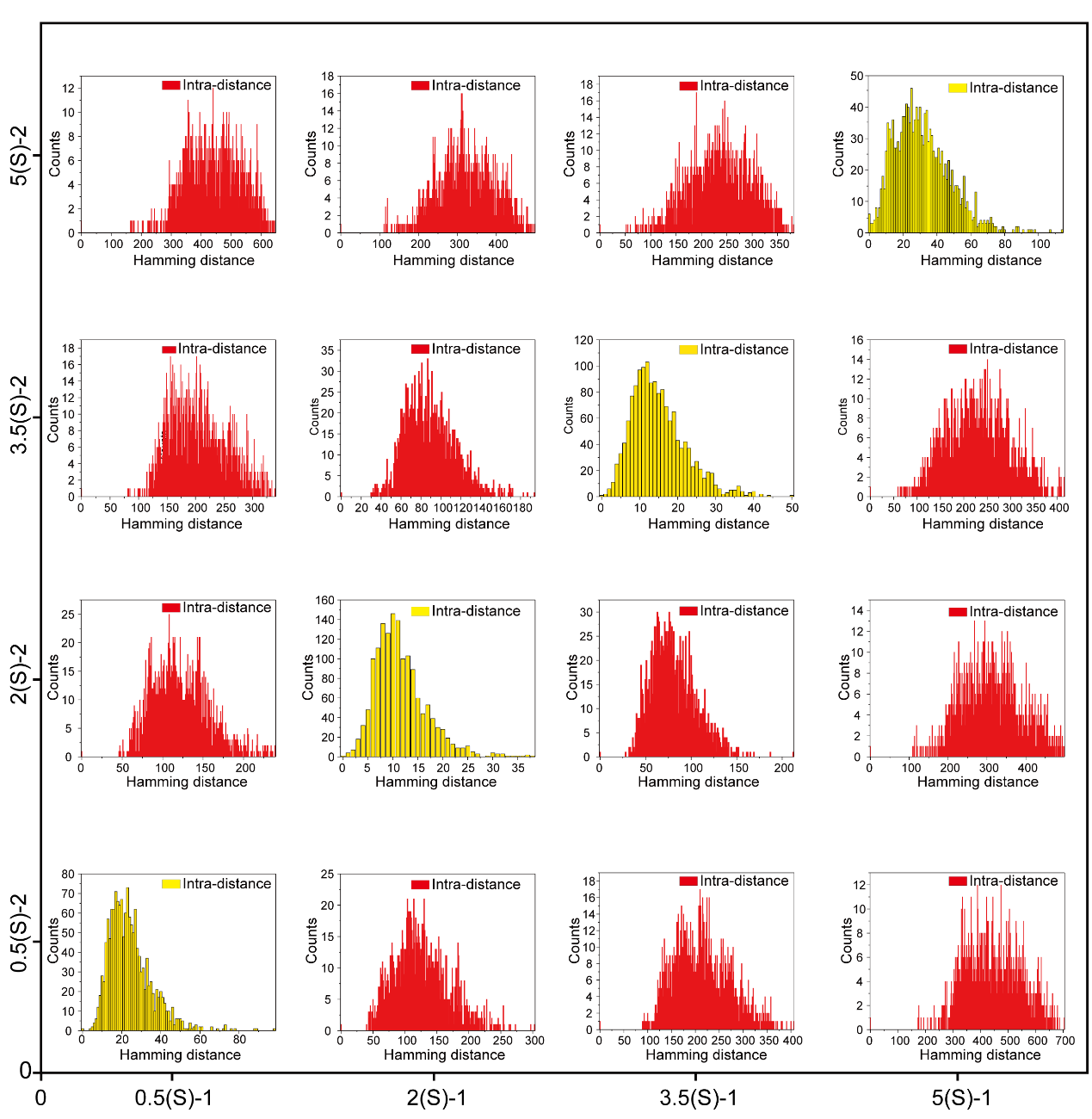


**Supplementary Fig. S16** Hamming intra-distance statistical image of binary coding that measuring the same PUF twice at four moments.


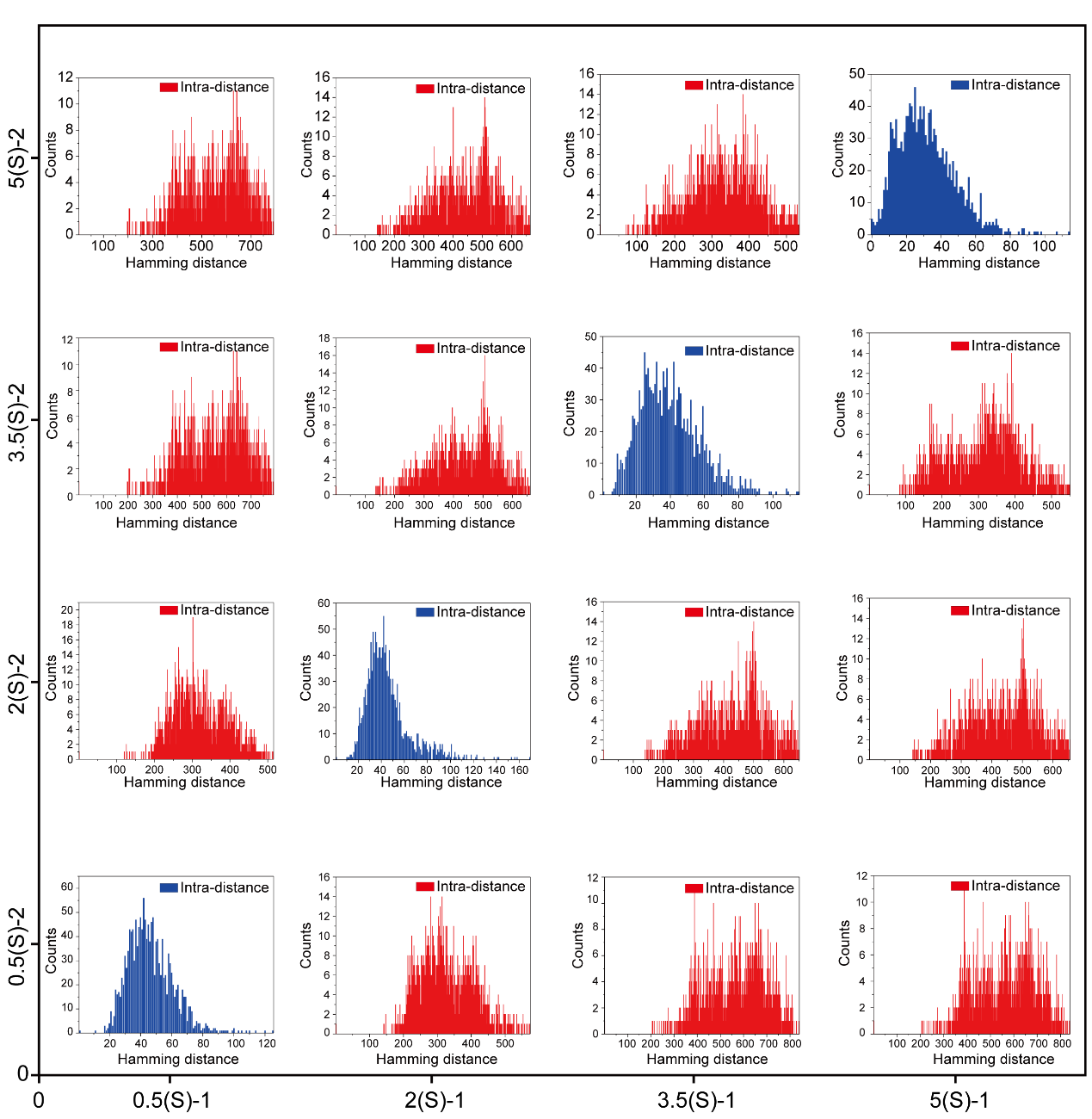


**Supplementary Fig. S17** Hamming intra-distance statistical image of quaternary coding that measuring the same PUF twice at four moments.


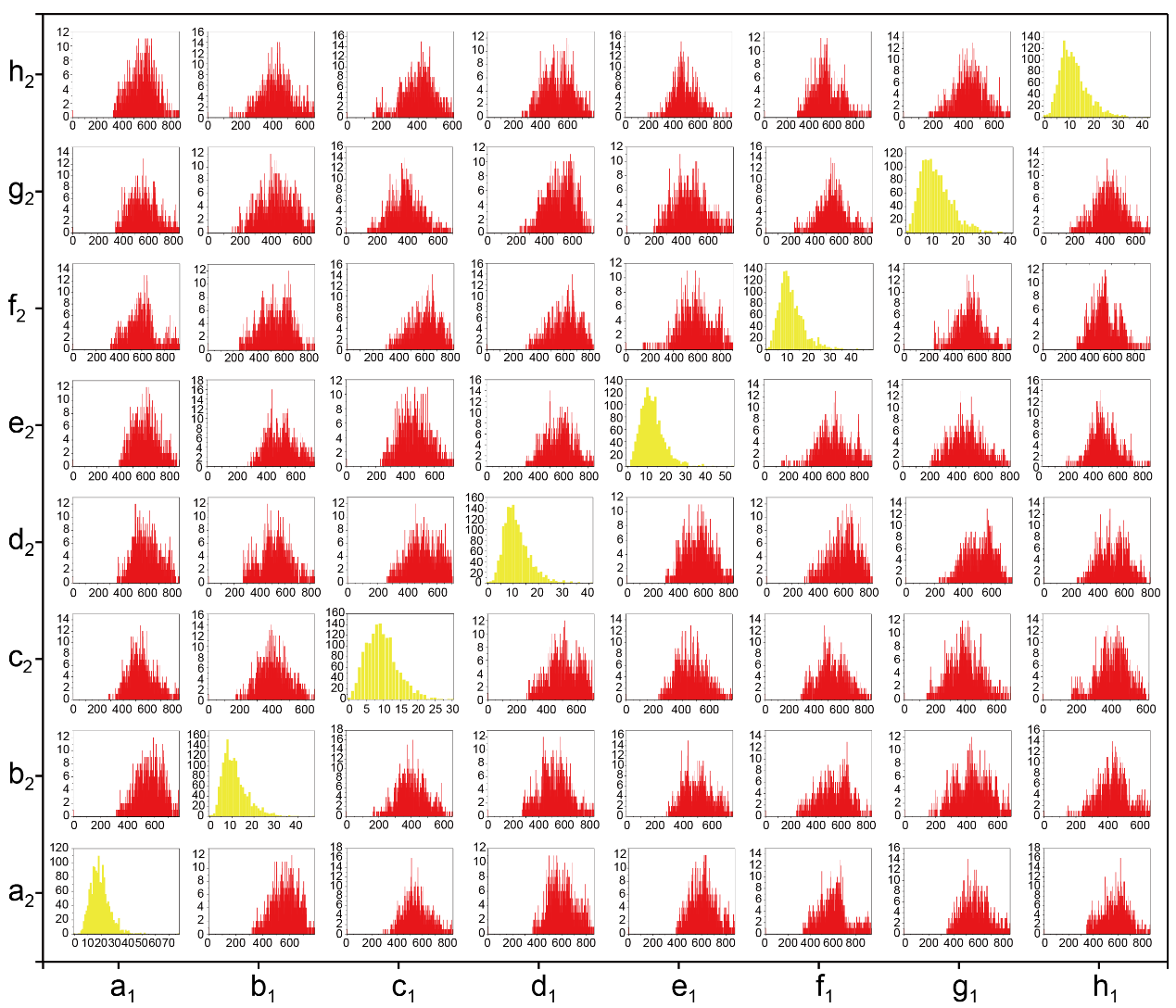


**Supplementary Fig. S18** Hamming intra-distance and inter-distance statistical maps of binary coding which eight PUFs were measured twice at the same moment (t = 0.5 s).


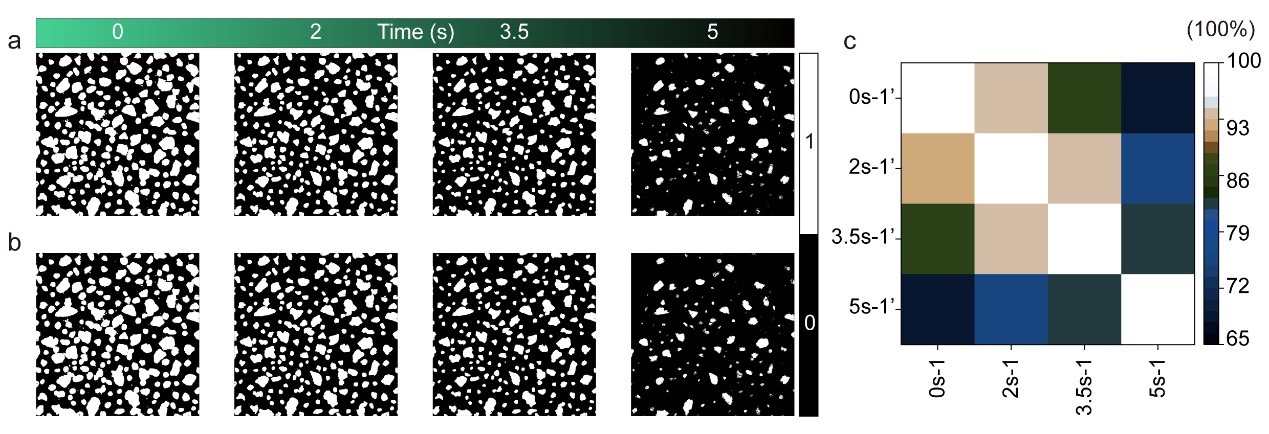


**Supplementary Fig. S19** The binary coding map of the TD-PUF measured for the first **a** and the second **b** time at four different times. **c,** Similarity statistical image of the TD-PUF with binary coding measured twice at four different moments.


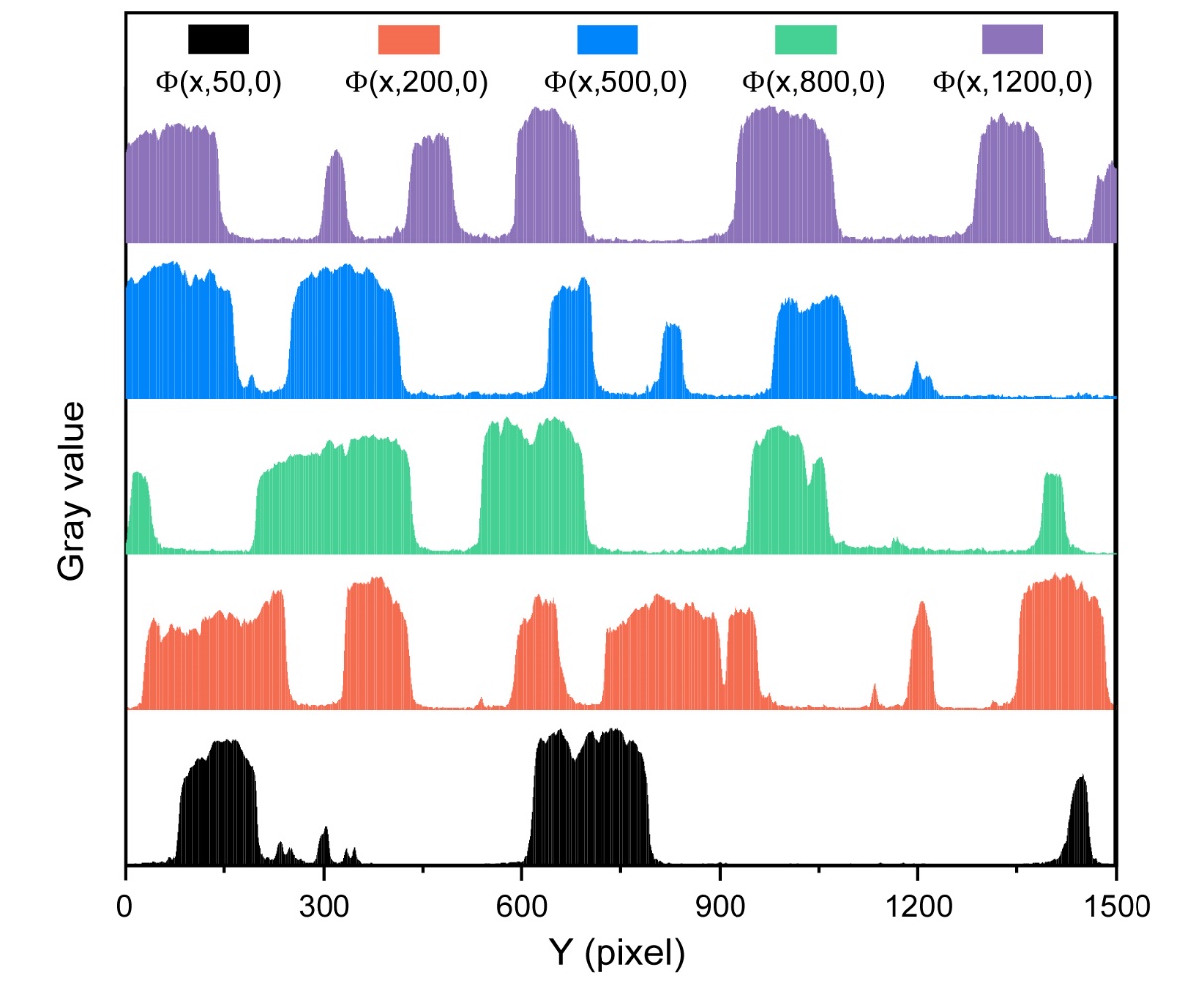


**Supplementary Fig. S20** The corresponding PMF ($\Phi$) of the TD-PUFs along (x, 50), (x, 200), (x, 500), (x, 800), (x, 1200) axes at t = 0 s.


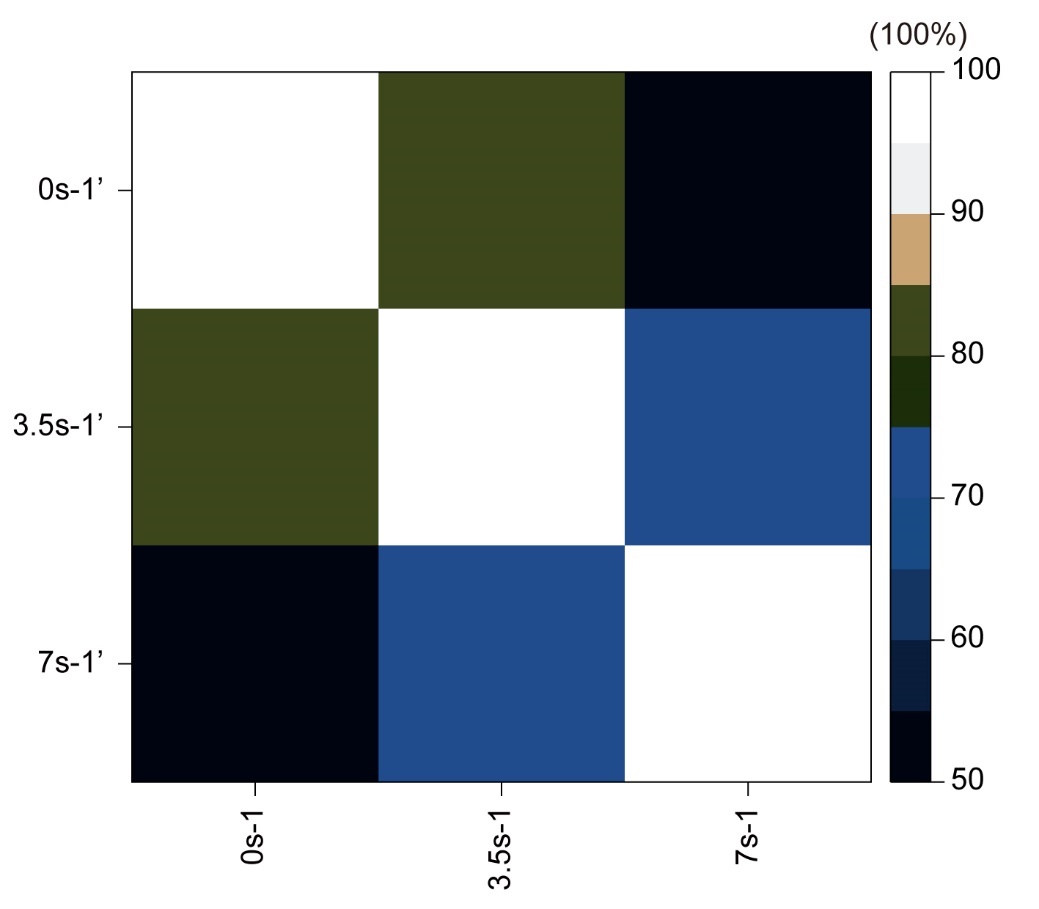


**Supplementary Fig. S21** The TD-PUFs’ similarity result after two repeated measurements at three different moments.

**Practical applications of TD-PUFs**

To demonstrate the practical applications of PUFs, we present two scenarios as depicted in Supplementary Fig. S18. First, CDs were affixed to double-sided tape to create PUF labels that can be applied to objects of complex shapes. Supplementary Fig. S18a shows an optical photograph of a PUF label attached to a medicine bottle. The time-varying phosphorescent response of this PUF label under UV light excitation is illustrated in Supplementary Fig. S18b. The corresponding quaternary coding and security performance analysis results are shown in Supplementary Fig. S18b (bottom) and Supplementary Fig. S19. Second, CD particles can be patterned into various PUF designs. For example, we created a PUF pattern featuring the initials of Zhengzhou University, as shown in Supplementary Fig. S18c. The time-varying phosphorescent response images of this pattern under UV excitation are displayed in Supplementary Fig. S18c (left), with the corresponding binary coding shown in Supplementary Fig. S18c (right). The similarity index of PUF patterns over time is provided in Supplementary Table 3. Despite the PUF being fixed in shape, the inherent randomness in the shape and relative position of CD particles ensures that the PUFs maintain a high level of randomness. Supplementary Fig. S18d illustrates the application and validation process of PUF labels within the supply chain. Initially, commodities with PUF labels are processed and manufactured by a plant. During this process, the PUF labels are read, digitized, and stored in a data cloud. These commodities are then shipped to various points of sale. Throughout the circulation process, end-users can read and verify the PUF labels to authenticate the commodities. Images of responses at three time points, defined as PUF_1_, PUF_2_, and PUF_3_, are shown in Supplementary Fig. S18d (left). PUF_2_ contains correct information, while PUF_1_ and PUF_3_ contain incorrect information. Thus, the binary coding of the Zhengzhou University icon (ZZU) must appear between 2-5 seconds (at 4 seconds) to

be correctly read by the end-user.


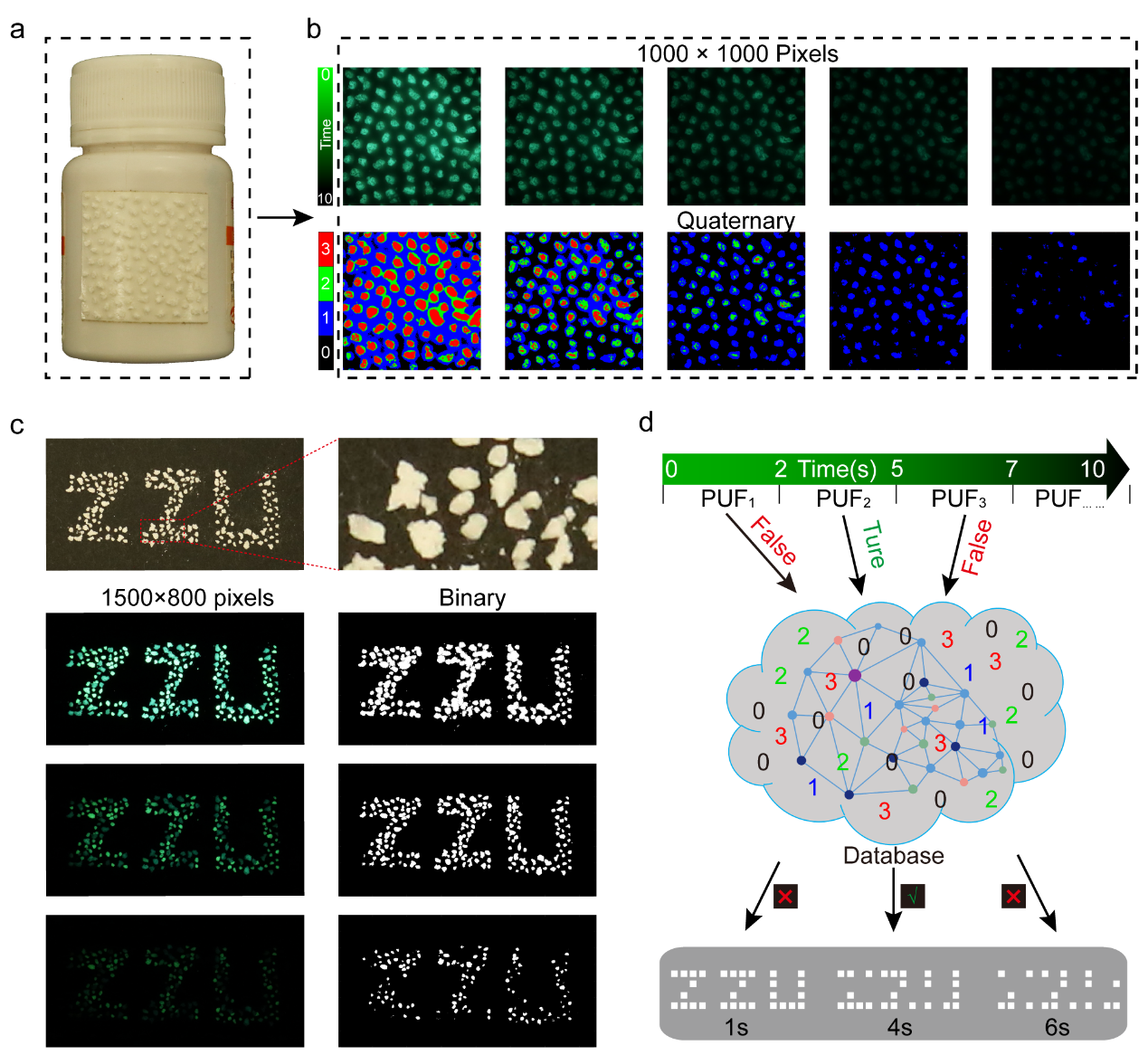


**Supplementary Fig. S22 a**, **b** Phosphorescence response images of PUF label pasted on drug bottle over time and corresponding quaternary coding. **c**, Phosphorescence response images of PUF pattern of Zhengzhou University (ZZU) over time and corresponding binary coding. **d**, Flow chart of the practical application of PUF labels in the supply chain.

**
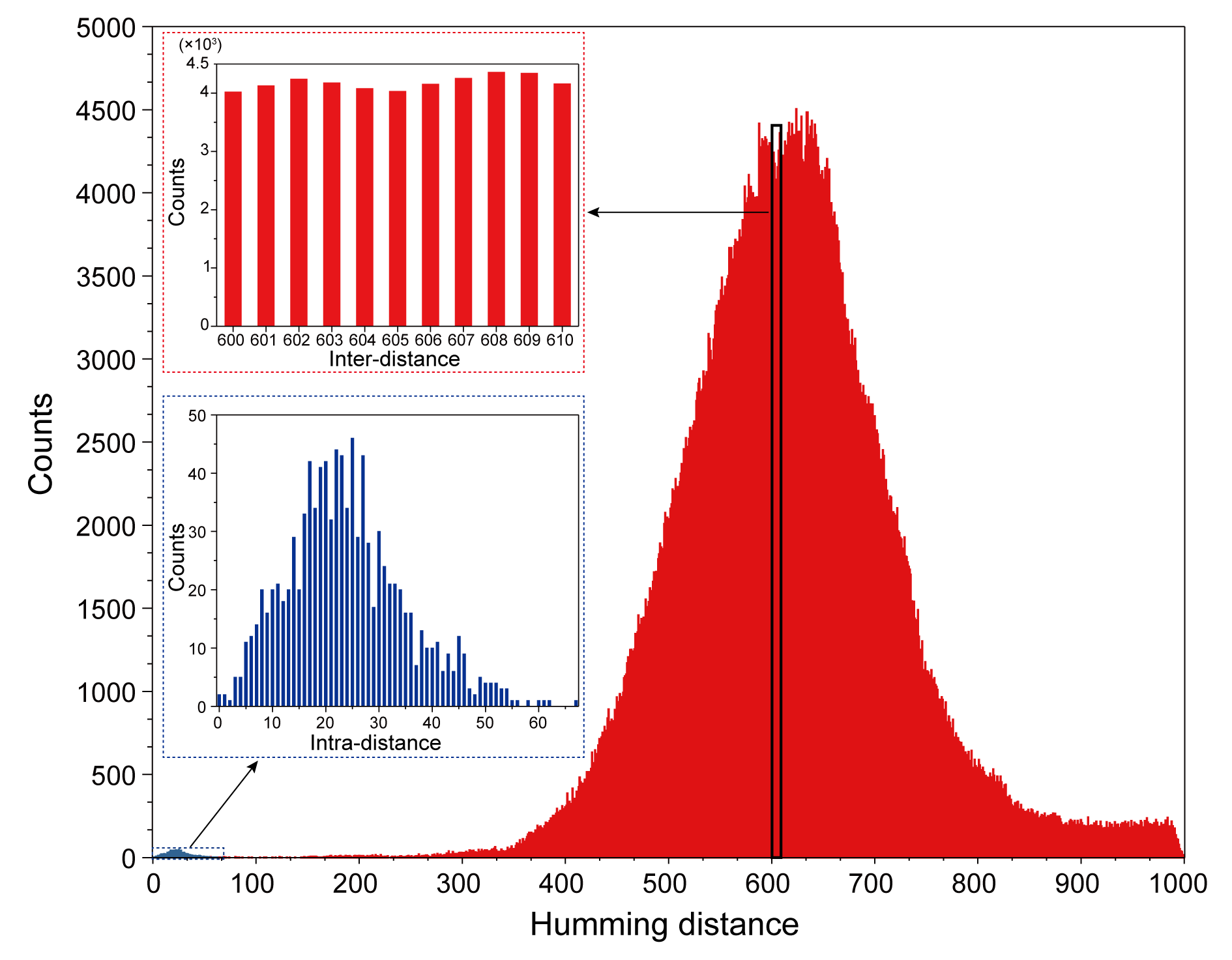
**

**Supplementary Fig. S23** Uniqueness and repeatability of the PUF label affixed to medicine bottle.


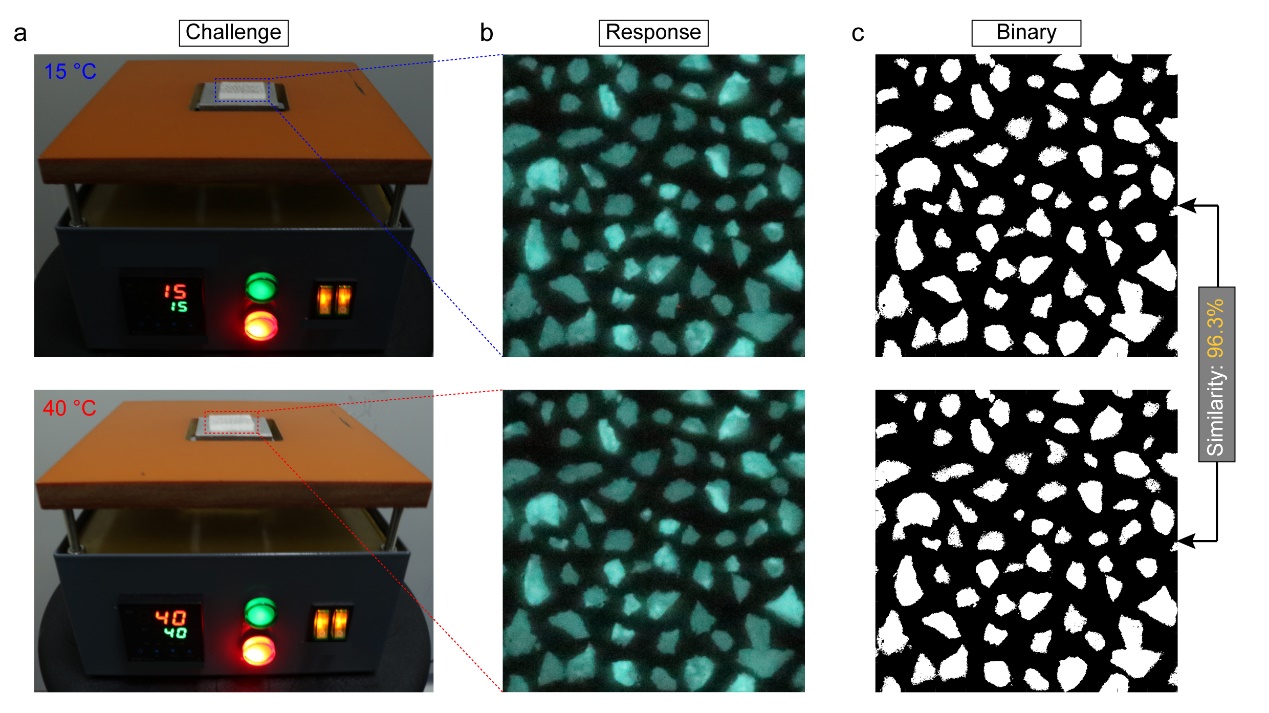


**Supplementary Fig. S24 a** Optical photos of TD-PUF treated on a heating platform at 15°C and 40°C. **b** Optical photos of the response of this TD-PUF at 15°C (top) and 40°C (bottom) under the challenge of 365nm UV light. **c** The corresponding binary coding images.


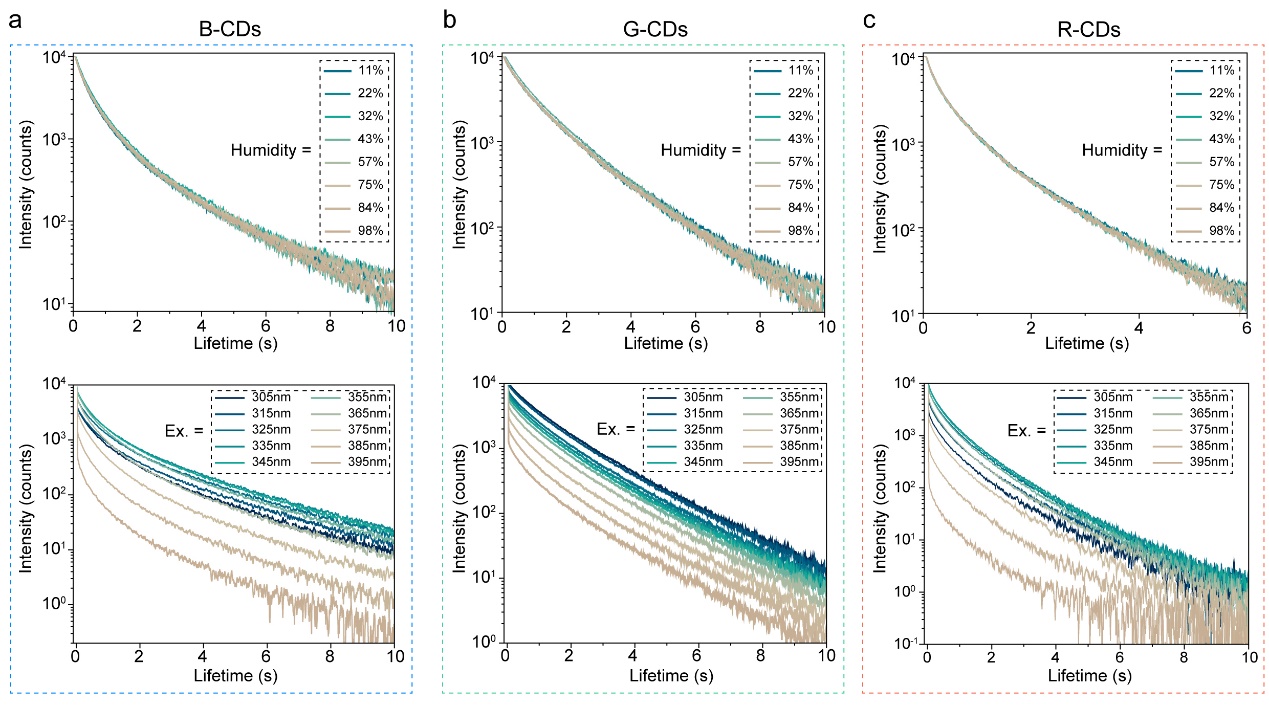


**Supplementary Fig. S25 a** The phosphorescence lifetime decay curves of the B-CD with humidity in the range from 11% to 98% (top) and excitation wavelengths from 305 nm to 395 nm (bottom). **b** The phosphorescence lifetime decay curves of the G-CD with humidity in the range from 11% to 98% (top) and excitation wavelengths from 305 nm to 395 nm (bottom). **c** The phosphorescence lifetime decay curves of the R-CD with humidity in the range from 11% to 98% (top) and excitation wavelengths from 305 nm to 395 nm (bottom).


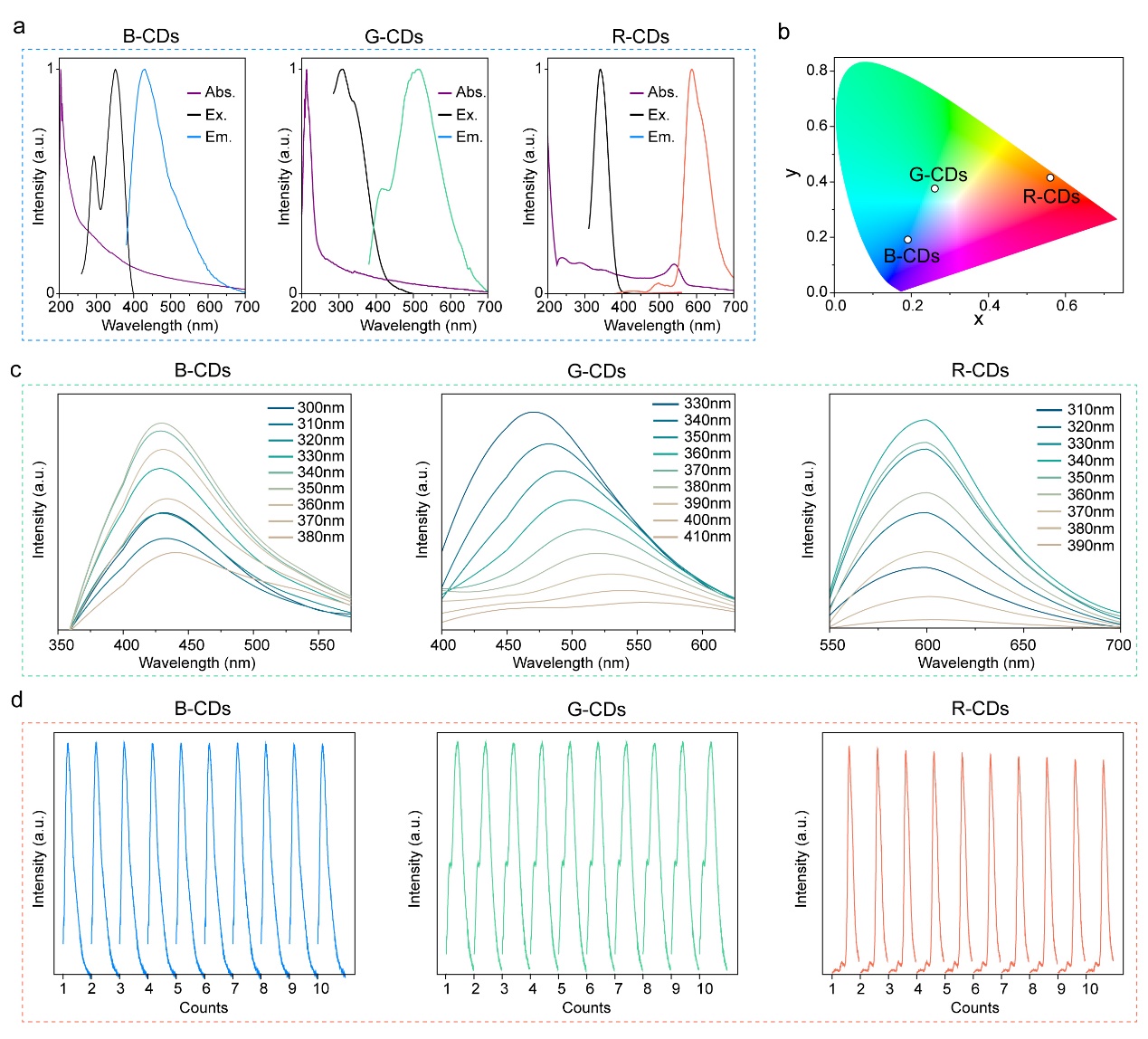


**Supplementary Fig. S26 a** Absorption, excitation and phosphorescence spectra of the B-CDs, G-CDs and R-CDs (from left to right). **b** Color coordinate of the B-CDs, G-CDs and R-CDs. **c** Phosphorescence spectra of the B-CDs, G-CDs and R-CDs under different excitation wavelengths (from left to right). **d** Phosphorescence intensity of B-CDs, G-CDs and R-CDs (from left to right) under 365 nm excitation for 10 minutes over ten cycles.


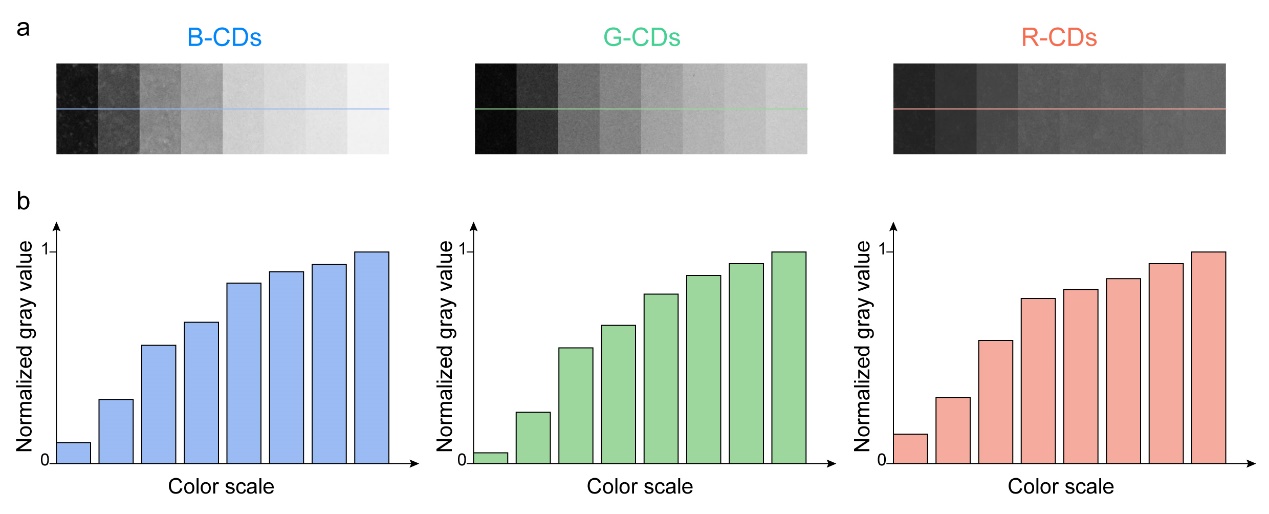


**Supplementary Fig. S27 a** The grayscale image corresponding to the color scale image prepared by the three CDs at different concentrations. **b** The statistical results of the average grayscale values of the color scale.


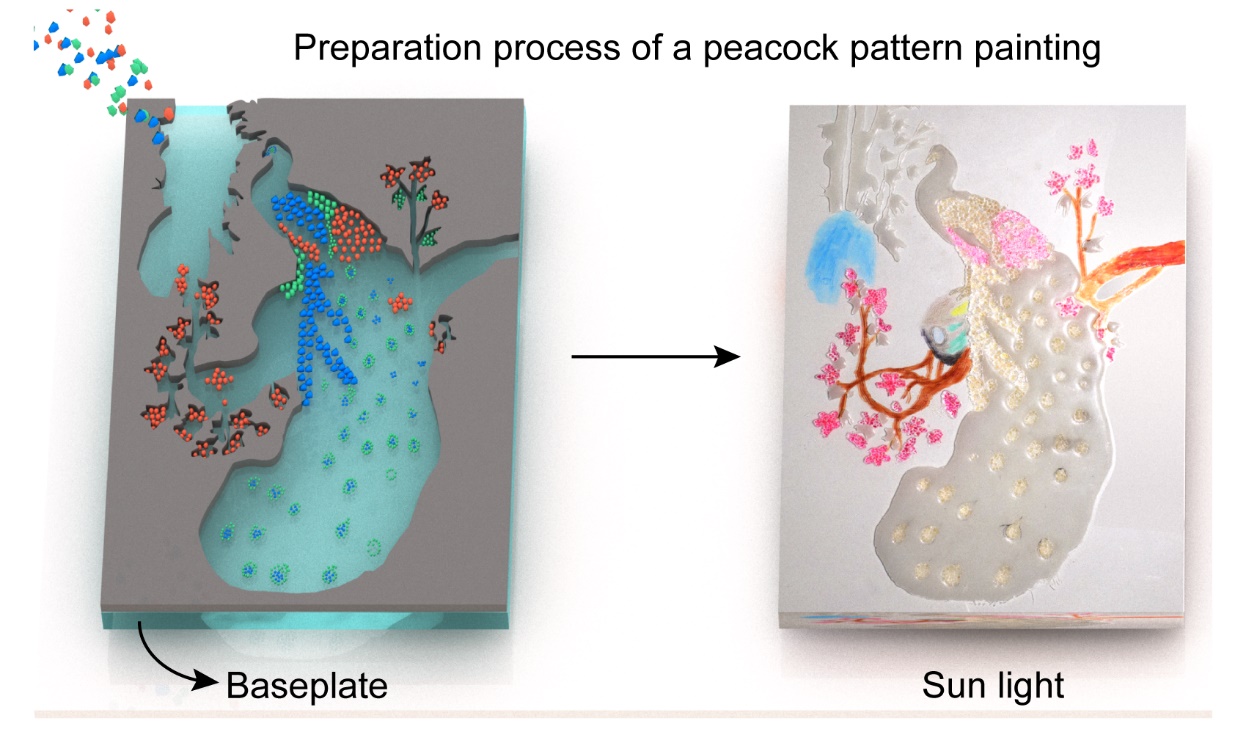


**Supplementary Fig. S28 a** Flow chart of preparing TD-PUF by mixing B-, G-, R-CD and randomly throwing them onto the surface with peacock pattern. **b** Corresponding optical photo of the real object.


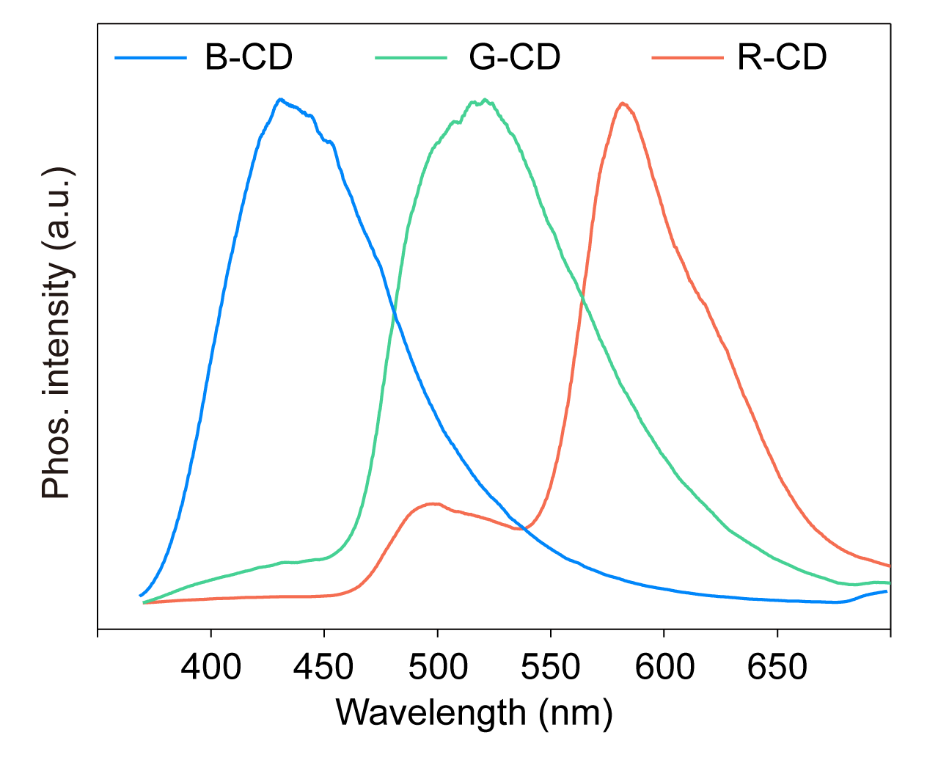


**Supplementary Fig. S29** Phosphorescence spectra of three CDs.


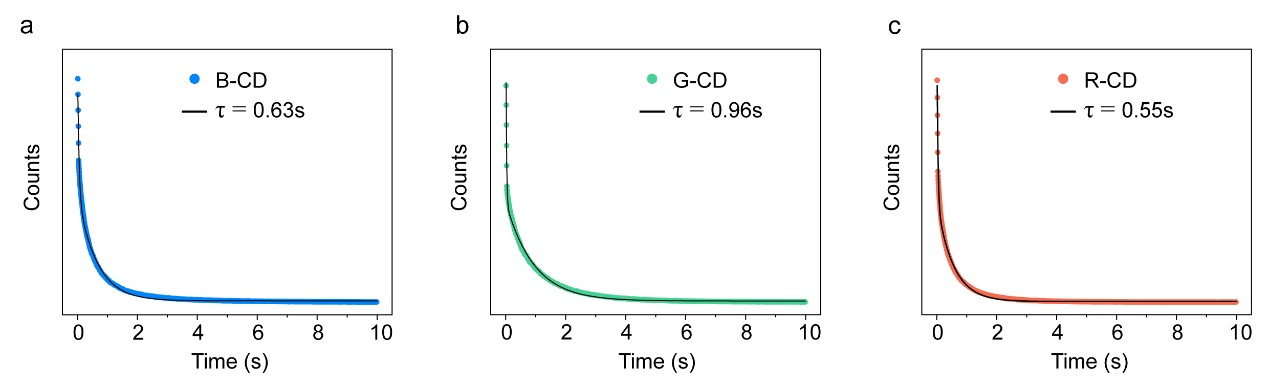


**Supplementary Fig. S30** Phosphorescence lifetime curves of **a** B-, **b** G, and **c** R-CDs.


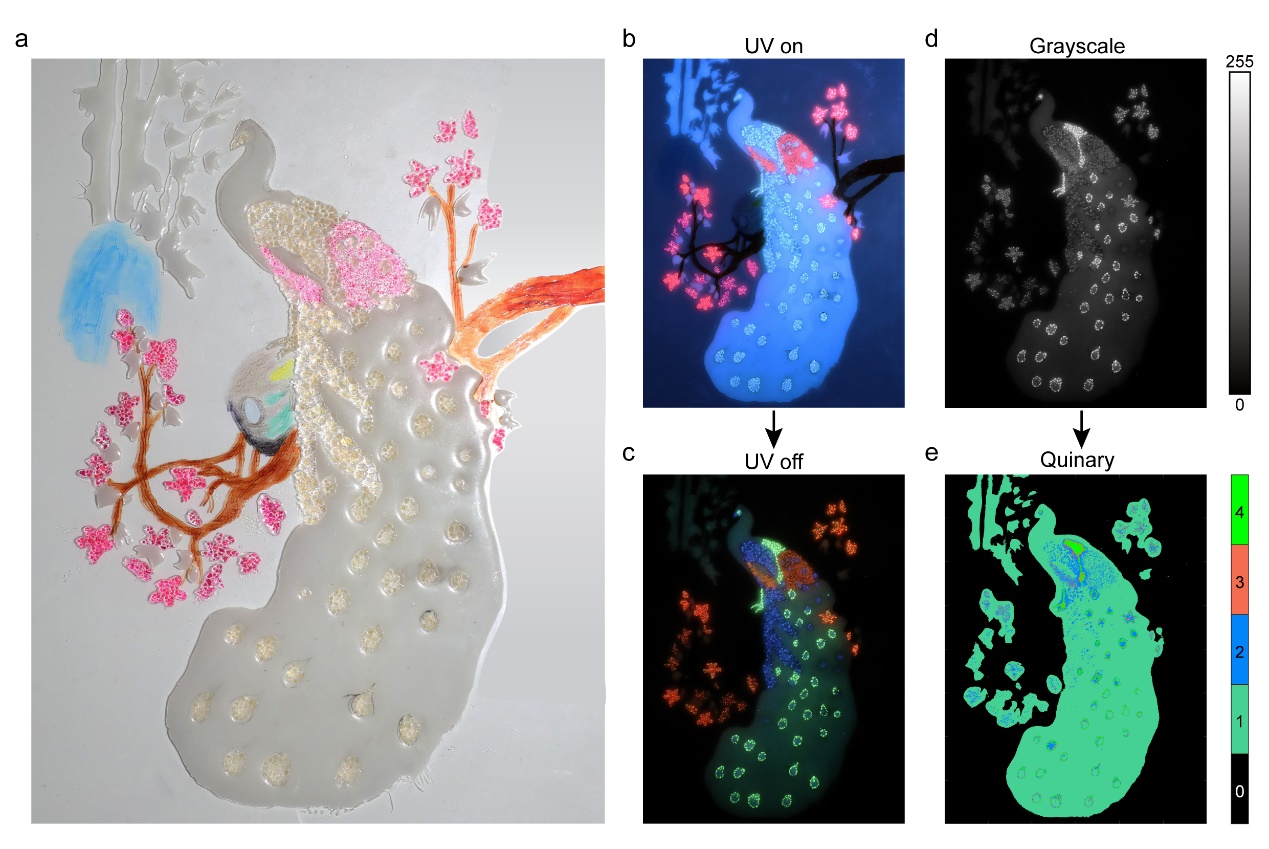


**Supplementary Fig. S31 a** Photos of TD-PUFs based on engraved peacock paintings under natural light, **b** photos excited by UV light, and **c** light photos after removing the UV light. **d** The corresponding grayscale image, **e** the corresponding quinary coding image.

**Supplementary Table S1** Coding capability of different pixel TD-PUFs.

| Resolution (pixels) | Coding capability | | |
| --- | --- | --- | --- |
|  | Binary | | |
|  | Information entropy | M | M’ |
| 10×10 | 0.80 | 2^100^ | 80 |
| 100×100 | 0.90 | 2^10000^ | 9000 |
| 1000×1000 | 0.95 | 2^1000000^ | 950000 |
| 1500×1500 | 0.87 | 2^2250000^ | 1957500 |

**Supplementary Table S2** Uniformity of different TD-PUFs.

| Name | Uniformity (%) | | | |
| --- | --- | --- | --- | --- |
|  | Binary | Quaternary | | |
|  | ‘1’ | ‘1’ | ‘2’ | ‘3’ |
| TD-PUFs-1 | 48.6 | 16 | 16.3 | 16.3 |
| TD-PUFs-2 | 50.6 | 17.1 | 16.3 | 17.2 |
| TD-PUFs-3 | 46.0 | 15.3 | 15.3 | 15.4 |
| TD-PUFs-4 | 52.8 | 18 | 16.4 | 18.4 |
| TD-PUFs-5 | 48.0 | 16.8 | 15.3 | 15.9 |

‘1’, ‘2’, ‘3’ represent the bit value of the binary and quaternary coding.

**Supplementary Table S3** The similarity index of PUFs with different pixels.

| Moment (s) | Similarity index (%) | | | |
| --- | --- | --- | --- | --- |
|  | Binary | | Quaternary | |
|  | I_11’_ | I_12_ | I_11’_ | I_12_ |
| 0 | 98.38 | 54.16 | 96.91 | 49.43 |
| 1.5 | 99.09 | 57.33 | 97.55 | 53.1 |
| 2 | 99.23 | 59.49 | 96.96 | 54.28 |
| 2.5 | 99.25 | 61.92 | 97.11 | 58.28 |
| 3.5 | 99 | 65.7 | 97.36 | 64.14 |
| 4 | 98.37 | 74.23 | 98.24 | 74.21 |
| 5 | 97.91 | 89.98 | 97.91 | 89.98 |

I_11’_ stand for similarity indexes between the first and the second measurement of the same PUF, I_12_ represents the similarity indexes between the first measurement of the PUF and another PUF, I_1’2_ represent the second measurement of the PUF and another PUF.

**Supplementary Table S4** The similarity index of two patterns’ PUF over time.

| Moment (s) | Similarity index (%) | | | |
| --- | --- | --- | --- | --- |
|  | PUF_1_ | | PUF_2_ | |
|  | Binary | Quaternary | Binary | Quaternary |
| 0 | 99.91 | 89.01 | 98.03 | 97.15 |
| 1.5 | 90.34 | 90.34 | 99.15 | 97.56 |
| 2 | 94.40 | 90.50 | 99.34 | 97.38 |
| 2.5 | 97.60 | 95.91 | 99.26 | 97.72 |
| 3.5 | 97.45 | 97.37 | 98.98 | 97.89 |
| 4 | 98.87 | 98.87 | 99.33 | 98.70 |
| 5 | 99.95 | 99.95 | 99.39 | 99.15 |
| 7 | 100 | 100 | 99.62 | 99.62 |

PUF_1_ stands for label attached to medicine bottle, and PUF_2_ stands for the PUF with ZZU pattern.

**Supplementary Table S5** Comparison between TD-PUFs and representative optical PUFs.

| **Representative Literature (DOI)** | **Stability** | **Pixel Count (Bits)** | **Reading Time** | **Encoding Capacity** |
| --- | --- | --- | --- | --- |
| 10.1002/adma.202102542 | Stable under controlled conditions; static reversible encoding | 480 | Seconds | 10^17^ |
| 10.1021/acsnano.3c12432 | Stable over a very short time window only | 896 | Seconds | 2^156250^ |
| 10.1038/s41467-019-14070-9 | PL intensity decay <5% over 90 days under ambient conditions | 2500 | Minute | 3 × 10^15051^ |
| 10.1038/s41467-024-55646-4 | <10% variation under temperature/humidity changes; long-term stability verified | 731471 | Seconds | 10^220322^ |
| 10.1038/s41467-024-47479-y | <5% drift over 60 days; response remains stable | 400 | Seconds | 3.8726 × 10^279^ |
| 10.1038/s41377-023-01285-1 | ~95% retention over 30 days | 5184 | Seconds | 9.54236×10^15^ |
| 10.1038/s41563-023-01734-7 | <5% intensity drop over 7 days; response is stable | 1,323 | Seconds | 2^1,750^ |
| **This Work (TD-PUFs)** | **High stability demonstrated under temperature, humidity, atmospheric, and long-term storage conditions (see Figs. R1–R4)** | **2250000** | **Seconds** | **16^225000^** |
